# Prognostic and clinicopathological significance of long noncoding RNA CTD-2510F5.4 in gastric cancer

**DOI:** 10.1007/s10120-018-00911-x

**Published:** 2018-12-17

**Authors:** Zhe Wang, Baoli Qin

**Affiliations:** Medical Oncology Department of Gastrointestinal Cancer (1), Cancer Hospital of China Medical University, Liaoning Cancer Hospital and Institute, NO.44 Xiaoheyan Road, Dadong District, Shenyang, 110042 Liaoning People’s Republic of China

**Keywords:** LncRNAs, Bioinformatics analysis, CTD-2510F5.4, Biomarker, Gastric cancer

## Abstract

**Background:**

Compelling studies have demonstrated the correlation between aberrant expressed lncRNAs and human cancers, and revealed promise of these lncRNAs as biomarkers in predicting patients’ survival and outcome.

**Methods:**

We downloaded the RNA-seq data from the Cancer Genome Atlas, and screened out DEGs and DELs between gastric cancer tissues and normal gastric tissues. By bioinformatics analysis, we identified CTD-2510F5.4 was a malignant phenotype associated lncRNA. The expression levels of CTD-2510F5.4 in tissues were detected by ISH, and the relationships between CTD-2510F5.4 expression and clinicopathological characteristics were analyzed by statistical analysis.

**Results:**

By bioinformatics analysis and functional analysis, we identified CTD-2510F5.4 was a malignant phenotype associated lncRNA of gastric cancer that potentially regulated cell cycle and apoptosis. CTD-2510F5.4 expression was significantly higher in gastric cancers, and was correlated with pathological grade, vascular or nerve invasion, AJCC TNM stage and OS. Moreover, gastric cancer patients with high CTD-2510F5.4 expression showed significantly shorter MST. High CTD-2510F5.4 expression was a independent risk factor for gastric cancers at pathological grade < III and without vascular or nerve invasion.

**Conclusions:**

We identified CTD-2510F5.4 was a malignant phenotype associated lncRNA potentially involved in the pathogenesis of gastric cancer. Our data also supported the clinical potential of CTD-2510F5.4 being a diagnostic and prognostic biomarker for gastric cancer.

**Electronic supplementary material:**

The online version of this article (10.1007/s10120-018-00911-x) contains supplementary material, which is available to authorized users.

## Introduction

High incidence and mortality rates of gastric cancer have made it a big concern for human public health worldwide, particularly in the developing countries [1]. 50% of gastric cancers were diagnosed in Eastern Asia, with majority cases in China [2]. The incidence and mortality rates of gastric cancer have steadily increased globally, and it was predicted that gastric cancer will become one of the top 15 leading causes of deaths among all disease in 2020 and 2030 [3]. The outlook for gastric cancer was poor as most patients already developed disseminated disease at the first time of diagnosis. This is probably due to the lack of non-invasive, early diagnostic tool [4].

Long noncoding RNAs (lncRNAs) were characterized as non-protein coding transcripts with a length of more than 200 nucleotides [5]. They fundamentally regulated gene expression via participating in molecular mechanisms including transcription, alternative mRNA splicing, translation and chromatin remodeling, therefore were closely related to the etiology of human disease [6]. Of particular interest, many published studies have identified the correlation between aberrant expressed lncRNAs and human cancers, and revealed promise of these lncRNAs as biomarkers in predicting patients’ survival and outcome [7–9]. In gastric cancer, overexpressed GAPLINC was found to be associated with poor patient outcome [10]. Similarly, high level of GClnc1 was also identified to be a predictor of poor prognosis for gastric cancer patients [11]. It displayed oncogenic characters by altering cancer cell invasion and proliferation via epigenetic mechanisms. Downregulation of certain lncRNAs such as FENDRR was also identified to be associated with poor prognosis [12].

In the present study, we downloaded the RNA-seq data of matched gastric cancer and adjacent normal tissue from the Cancer Genome Atlas (TCGA) data portal, and screened out the DEGs and DELs. Weighted Gene Co-Expression Network Analysis (WGCNA) was performed to identify gene and lncRNA modules associated with clinical traits. By examining interacting gene set and pathways enrichment, as well as co-expression networks, we identified CTD-2510F5.4 was a key lncRNA potentially involved in the molecular pathogenesis of gastric cancer via regulating cell proliferation. Functional analysis also revealed the regulatory roles of CTD-2510F5.4 in mediating cell cycle and apoptosis. In the analysis of the correlation between CTD-2510F5.4 expression level and gastric cancers, we found the presence of high CTD-2510F5.4 expression in gastric cancer tissues was correlated with clinicopathological characteristics. Furthermore, high CTD-2510F5.4 expression was also an independent factor for gastric cancers at pathological grade < III or without vascular or nerve invasion that related to shorter MST. These data supported the clinical potential of CTD-2510F5.4 being a diagnostic and prognostic biomarker for gastric cancer.

## Materials and methods

### Screening for differentially expressed genes (DEGs) between gastric cancer tissues and adjacent gastric tissues

RNA-seq data of gastric cancer were downloaded from TCGA data portal [13]. A total of 407 gastric cancer tissues and adjacent non-tumorous gastric tissues, including 27 matched pairs were recruited. Gene expression profiles for 19069 coding genes and 14448 lncRNA were obtained. The featured genes/lncRNAs were chosen based on the criteria as follows:

(1) Paired *t* test analysis with *p* value < 0.01; (2) gene median expression in gastric cancer > 0, and in adjacent-normal tissue > 0; (3) median ratio of expression level in gastric cancer against adjacent-normal tissue > 2 or < 0.5.

### Enrichment analysis of the DEGs

Gene ontology (GO) analysis and Kyoto Encyclopedia of Genes and Genomes (KEGG) pathway enrichment analysis were performed using R package cluster Profiler to observe the functions of the DEGs, each functional module and hub genes.

### Construction of gene co-expression network between DEGs and DELs

The R package WGCNA was used to build scale-free co-expression network for the hub DEGs and DELs. Gene expression similarity matrix was established by calculating Pearson correlation coefficient between two genes/lncRNAs, transformed into adjacency matrix (a threshold power of β = 5), and then into topological matrix. Topological overlap measure (TOM) was used to describe the degree of association between genes/lncRNAs. Defined as the first principal component for a module, module eigengene (ME) was calculated to indicate the overall level of gene expression within the module. A hub gene/lncRNA was selected based on the criteria of its module membership (MM) value being over 0.9.

### Gene–gene–lncRNA co-expression network analysis

The Search Tool for the Retrieval of Interacting Genes (STRING) database was used to annotate functional interactions for hub genes, and to construct the gene–gene–lncRNA network by recruiting functional relevant lncRNAs, with the purpose of observing the regulatory relationships between each lncRNA and the hub genes.

### Cell culture

Gastric cell line HGC-27 was maintained in complete growth medium of Roswell Park Memorial Institute medium (RPMI, Invitrogen, China), supplemented with 10% fetal bovine serum (FBS, Invitrogen, China) at 37 °C in a humidified incubator with 5% CO_2_.

### Quantitative real-time PCR (qRT-PCR)

cDNA was synthesized by GoScript™ Reverse Transcription System (Promega, USA). Amplification was performed in 20 µl reaction mixture containing 10 µl GoTaq® qPCR Master Mix (Promega, USA), 0.2 µl CXR Reference Dye, 2 µl primer and cDNA template (100 ng). Primers for all genes were: CTD-2510F5.4 (F: GGTCTCTTGCTCTGTCACCC; R: GCACACCTGTAGTCCCAGTT); GAPDH (F: CCAGCAAGAGCACAAGAGGAAGAG; R: GTCTACATGGCAACTGTGAGGAG); ATAD2 (F: CAACTTGCTAATGGCAGGCA; R: AGCCCTCAATGACCGAGTAAC); BUB1 (F: CATGAGGATCTGCCCGCTTC; R: CTGGAAGACATGGCGCTCTC); DTL (F: CTGGCGCTTGAATAGAGGCT; R: GGATGGATTGCACTTTACCC); KIF18B (F: CGTTCTAAGCAGTTGGCCCT; R: AGCTGCTGTAGGGTCTCAAAC); KPNA2 (F: CAGGAAAACCGCAACAACC; R: GGCAGCTTGAGTAGCTTGGAG); MCM10 (F: AACATGCTTTTCTGCGGAGC; R: TCGTCTGTAGGGGTTGGGAG); NUSAP1 (F: ACTGCAATCACTACTCCAAACTT; 5′ CAGTTCATTCATGGAATTGTGTTCT); RAD54L (F: GAGACCTTCCGCCTTCATGT; R: CTGTCCAGGGCTTGGTAAGT); RBL1 (F: GGACATCTTCCCCTGATGCC; R: GCGGTAGGAGAACTGTAGCG); E2F3 (F: TGATCCAAAGCTGCACCCTG; R: CTGGAGGGGCTTTCACAACT).

### siRNA transfection

Cells were transfected by X-tremeGENE siRNA Transfection Reagent (Sigma, China) according to the manufacturer’s instructions. CTD-2510F5.4 siRNA sequences were:

siRNA1:5′-GGAGUGGCAGUGUUGCAAUTT-3′.

siRNA 2: 5′-CCAGGCUGUAUUACUGUCUTT-3′.

### Cell viability assay

Cell viability was measured using the Cell Counting Kit-8 (CCK-8) (Shanghai Obio Technology, China) following the manufacturer’s instructions. Cells were seeded at a density of 5 × 10^3^ cells/well in 96-well plates, and the cell viability (O.D.) was calculated by measuring the absorbance at a wavelength of 450 nm. All assays were repeated at least three times.

### Flow cytometry

For apoptosis and necrosis analysis, HGC-27 cells were stained with the Annexin V-FITC Apoptosis Detection Kit (KeyGEN biotech, Jiangsu, China). For cell cycle analysis, the cells were fixed with 70% cold ethanol overnight, and stained with the Cell Cycle Detection Kit (KeyGEN biotech, Jiangsu, China). Experiments were repeated at least three times.

### Tissue microarray (TMA)

TMA were purchased from Shanghai Biochip Company Limited. Tissues embedded in the TMA were obtained from 90 gastric cancer patients with an average age of 67 (44–86 years), including 68 males (75.6%) and 22 females (24.4%). Tissue collection was approved by the Ethics Committee of Taizhou hospital of Zhejiang Province.

### In situ hybridization (ISH)

TMA blocks were sectioned at 4 µm thickness and mounted on coverglasses coated poly-l-lysine. After deparaffinized in xylene, rehydrated through a graded ethanol series, and incubated with 3% hydrogen peroxide to block endogenous peroxidase activity, TMA sections were treated with 3% citric acid diluted pepsin for at 37 °C 15 min and hybridized with CTD-2510F5.4 oligonucleotide probes (probe1: CACTGCAACCTCTGCCTCCCAGGTTCAAGTAACT; probe 2: GTCTCGCTATGTTTCCCAGGCTGTATTACTGTCTT; probe 3: TGCTATGGACTTCAGAGATTCCTTGGCAAGGCATTGTCGA) at 37 °C overnight. Sections were visualized followed by counterstaining with hematoxylin.

Expression level of CTD-2510F5.4 (%) was evaluated quantitatively and scored as 1 (negative), 2 (moderate positive) and 3 (strong positive). We randomly selected 10 fields with approximately 100 cells/field at a magnification of 400×, and recorded the staining score, frequency and percentage. The overall CTD-2510F5.4 expression level was calculated by multiplying staining score by mean value of staining percentage (0–300%).

### Statistical analysis

SPSS 19.0 and GraphPad Prism 7 were used for statistical analysis, and *P* < 0.05 was considered as statistically significant. Receiver operating characteristic (ROC) curve was used to determine the Cut Point value. Wilcoxon signed-rank test was used to compare expression level of CTD-2510F5.4 between gastric cancer and gastric tissues. Pearson *χ*^2^ test was used to determine the association between CTD-2510F5.4 expression and clinicalpathological characteristics. Non-conditional logistic regression was used to calculate the odds ratio (OR) and 95% confidence interval (CI) for analyzing the independent risk factors. The Kaplan–Meier model was used to analyze the correlation between high CTD-2510F5.4 expression and prognosis of gastric cancers.

## Results

### Identification of DEGs and DELs between gastric cancer tissues and normal tissues and enrichment analysis of DEGs

**Fig. 1:**
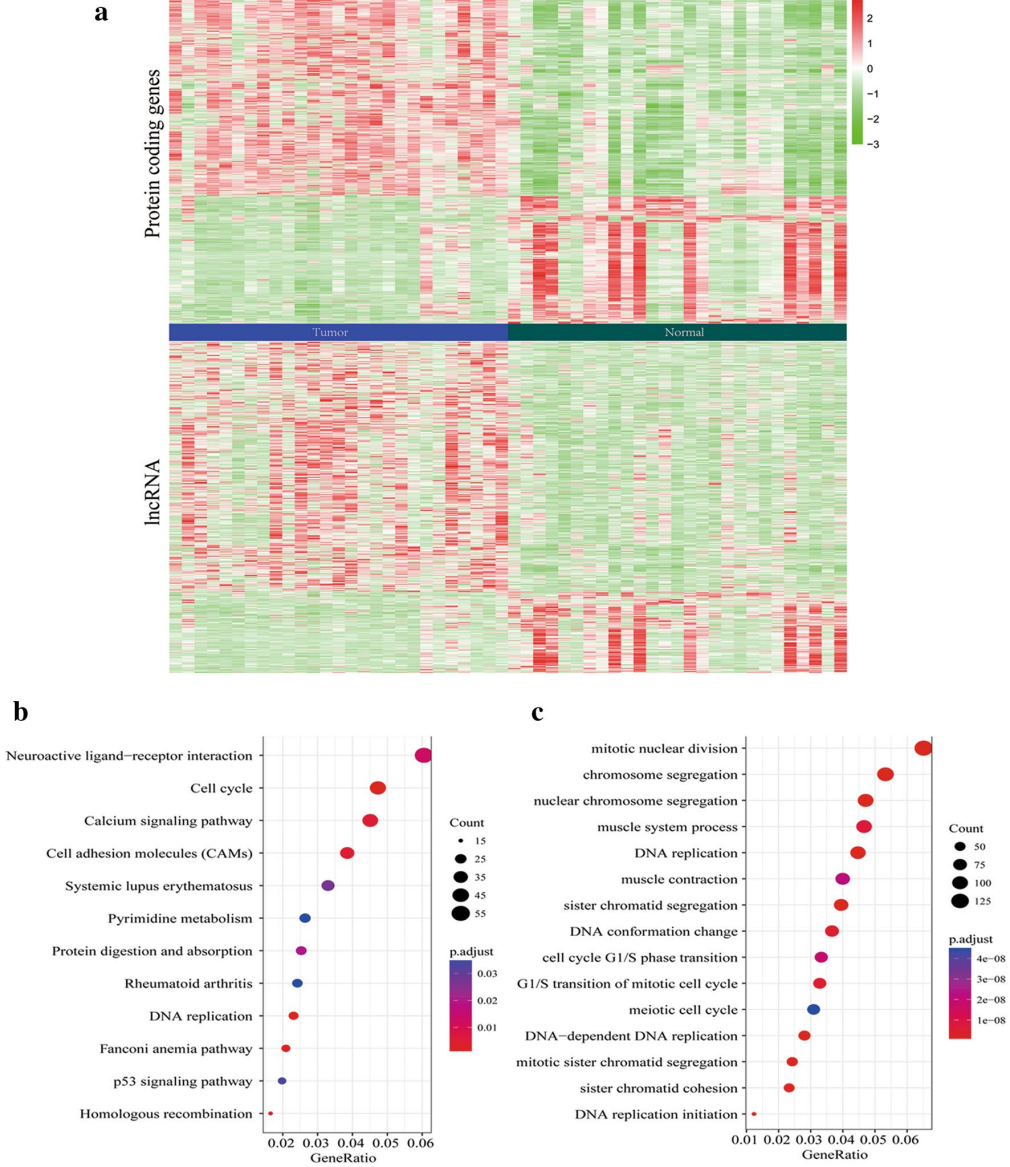
Heat map for coding genes and lncRNAs, as well as gene enrichment analysis. **a** Heat map showing gene expression profiles of 19069 coding genes and 14448 lncRNAs in 407 gastric cancer tissues and adjacent non-tumorous gastric tissues. *x*-axis indicated samples, and *y*-axis indicated genes. KEGG (**b**) and (**c**) biological processes of GO analysis to predict the potential roles of identified DEGs

Having obtained the gene expression profiles of 19069 coding genes and 14448 lncRNAs in 407 gastric cancer tissues and adjacent non-tumorous gastric tissues, we identified 2386 DEGs and 985 DELs based on the gene/lncRNA selection criteria. Cluster analysis (Fig. 1a) suggested that the expression profiles of identified DEGs and DELs can be distinguished between the gastric cancer tissues and the normal gastric tissues.

GO and KEGG pathway analysis was performed to predict the potential roles of identified DEGs (Table S1–S4). As shown in Fig. 1b, KEGG analysis showed these DEGs were mostly enriched in pathways including cell cycle, calcium signaling pathway, cell adhesion molecules (CAMs) and DNA replication. Biological processes of GO analysis (Fig. 1c) showed the enrichment of DEGs in mitotic nuclear division, DNA replication, cell cycle G1/S phase transition, G1/S transition of mitotic cell cycle, meiotic cell cycle, DNA-dependent DNA replication, and DNA replication initiation, etc. It is noteworthy that both analytic methods have indicated the enrichment of these DEGs in tumor-related pathways such as cell cycle regulation and DNA replication, supporting the potential participation of these DEGs in regulating tumor development and progression.

### Construction of co-expression network between DEGs and DELs

**Fig. 2:**
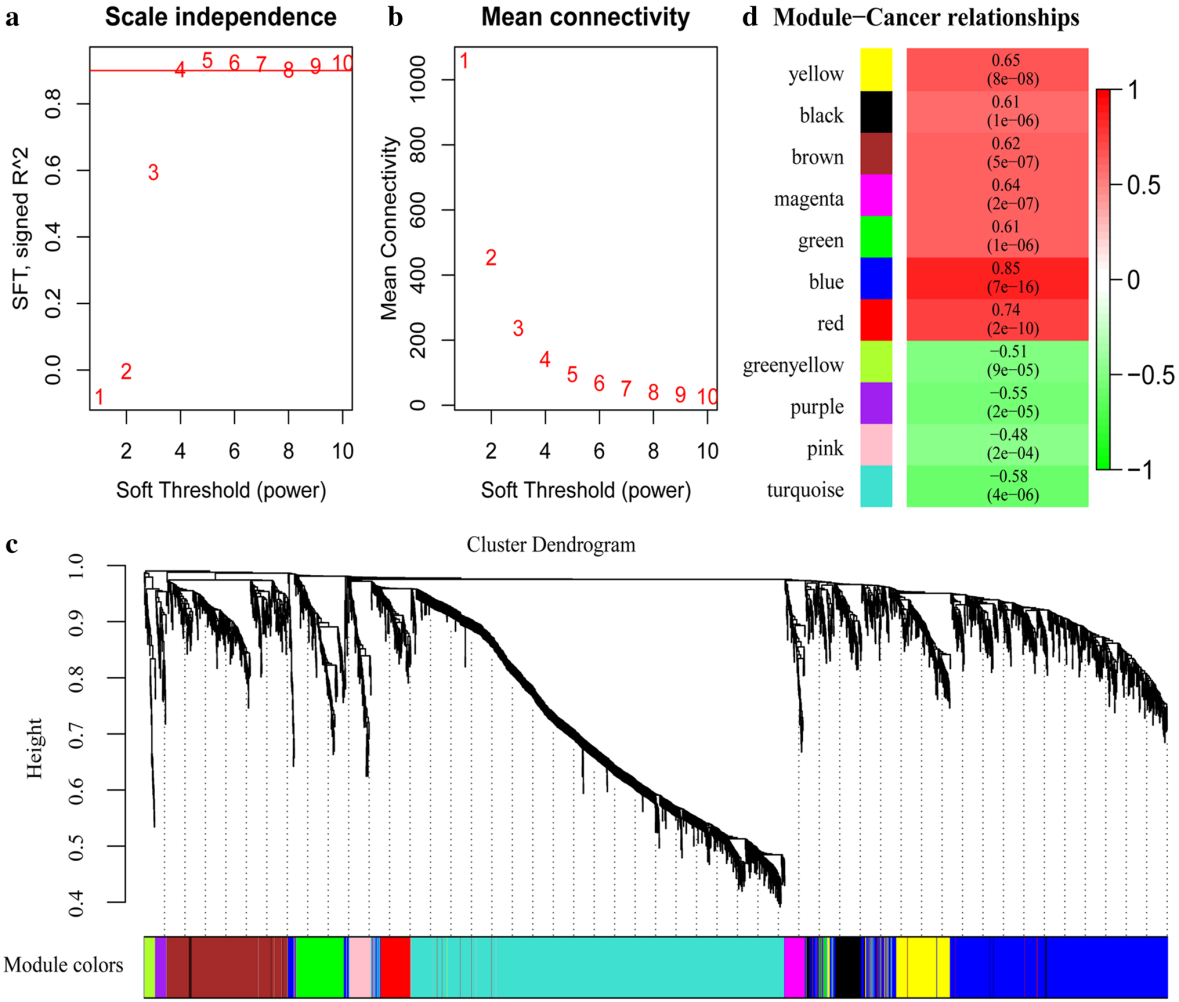
WNCNA analysis of DEGs and DELs. **a, b** Network topology analysis for powers of soft-thresholding; **c** dendrogram of DEGs and colors of all 11 modules; **d** correlation analysis for 11 modules and clinical traits. The numbers indicated the correlation coefficients and *p* values

WGCNA was exploited to cluster closely co-expressed DEGs and DELs into co-expression networks. We clustered these DEGs and DELs by average-linkage hierarchical clustering analysis by transforming adjacency matrix into TOM, and set each network module with a minimum of 30 genes/lncRNAs based on Dynamic Tree Cut standard (Fig. 2a, b). The eigengenes for each module were then calculated, and 11 new modules were generated on the basis of correlation efficiency (Fig. 2c). Genes in yellow, black, brown, magenta, green, blue and red modules were associated with gastric cancer tissues, while genes in green, yellow, purple, pink and turquoise modules were associated with normal tissues. Notably, the gray module was unable to be clustered into other modules. A total of 2374 DEGs and 979 DELs were allocated into 11 modules and the information of these DEGs and DELs were listed in Table S5.

The association between each module and clinical traits was calculated by the Pearson’s correlation coefficient between ME and sample traits (Fig. 2d). DEGs and DELs clustered in blue module showed the strongest correlation with gastric cancer, suggested blue module was gastric cancer highly correlated module.

### Screening for key modules and hub genes

Pathway enrichment analysis was performed on gene sets in each module (Table S6), and 7 modules were enriched in 64 KEGG pathways (Fig. S1). Distinct enriched pathways were displayed between different modules, inferring the independent functional notes of each module. It is notable that 6 of 11 pathways (54.5%) enriched in the blue module, such as cell cycle, DNA replication, homologous recombination, and p53 signaling pathway, were the same as we have identified for all DEGs.

We selected blue module as the key module depends upon the analytic results from the enrichment analysis and the module–cancer interactions. 15 hub genes were identified by way of calculating the correlation coefficients between genes and MEs in the blue module (Table S7). It was interesting again to find most of these genes were enriched in the process of cell replication and cell cycle regulation (Table S8).

### Construction of gene–gene–lncRNA network

Aiming for observing the correlation between lncRNA and hub genes in co-expression modules, we first determined the hub genes related lncRNAs by calculating the correlation coefficients between individual lncRNA and the hub genes. 5 lncRNAs of CTD-2510F5.4, RP11-120D5.1, RP5-991G20.1, DLEU2 and AC015849.16 were ultimately screened out.

**Fig. 3:**
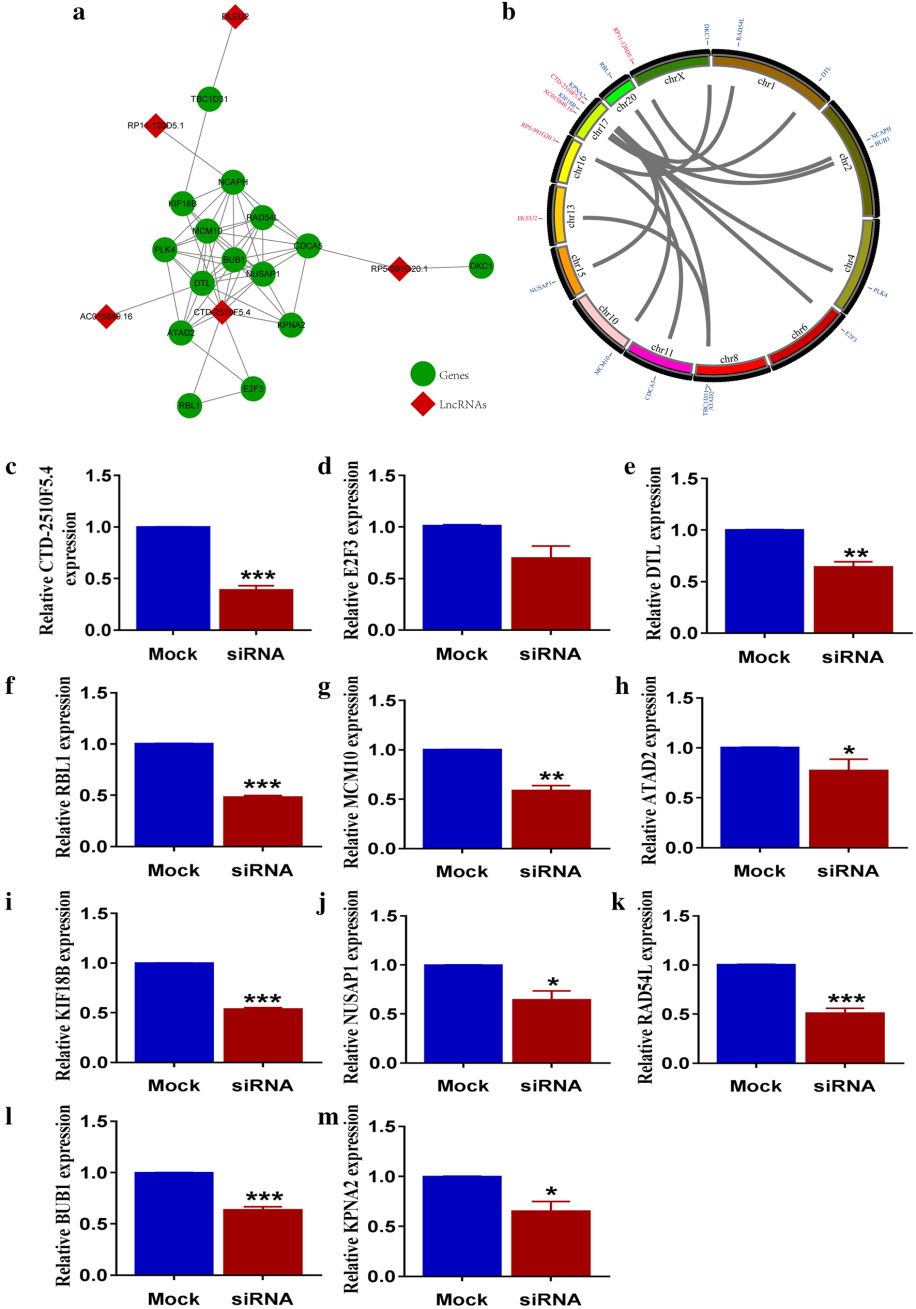
Interactions between hub genes and relevant lncRNAs. **a** Construction of gene–gene–lncRNA network; **b** chromosomal location of hub gene and associated lncRNAs; relative expression of CTD-2510F5.4 (**c**) and 10 hub genes E2F3 (**d**), DTL (**e**), RBL1 (**f**), MCM10 (**g**), ATAD2 (**h**), KIF18B (**i**), NUSAP1 (**j**), RAD54L (**k**), BUB1 (**l**), KPNA2 (**m**) after CTD-2510F5.4 knock down. The mRNA expression level of all genes was normalized against the endogenous control of GAPDH

We then generated the interaction network between these hub genes using STRING online database, and constructed gene–gene–lncRNA network after recruiting identified lncRNAs. As shown in Fig. 3a, CTD-2510F5.4 was closely related to 10 hub genes: E2F3, DTL, RBL1, NUSAP1, ATAD2, KIF18B, MCM10, RAD54L, BUB1 and KPNA2. Figure 3b indicates CTD-2510F5.4 possibly regulated these 10 hub genes in *trans*.

To address the modulatory role of CTD-2510F5.4 on these 10 hub genes, we knocked down CTD-2510F5.4 by siRNA transfection strategy. As shown in fig. S2, siRNA1 effectively reduced more than 60% of CTD-2510F5.4 expression after 48 h of transfection (*p* < 0.0001), whereas siRNA2 only reduced about 30% of gene expression (*p* < 0.0001). The mRNA expression level of 10 CTD-2510F5.4-related hub genes was then measured by qRT-PCR. In results, reduction in CTD-2510F5.4 expression significantly decreased expression of 9/10 hub genes (Fig. 3c–m), implying CTD-2510F5.4 could regulated these hub genes in gastric cancer cells.

### CTD-2510F5.4 knock down significantly reduced cell viability of gastric cancer cells

**Fig. 4:**
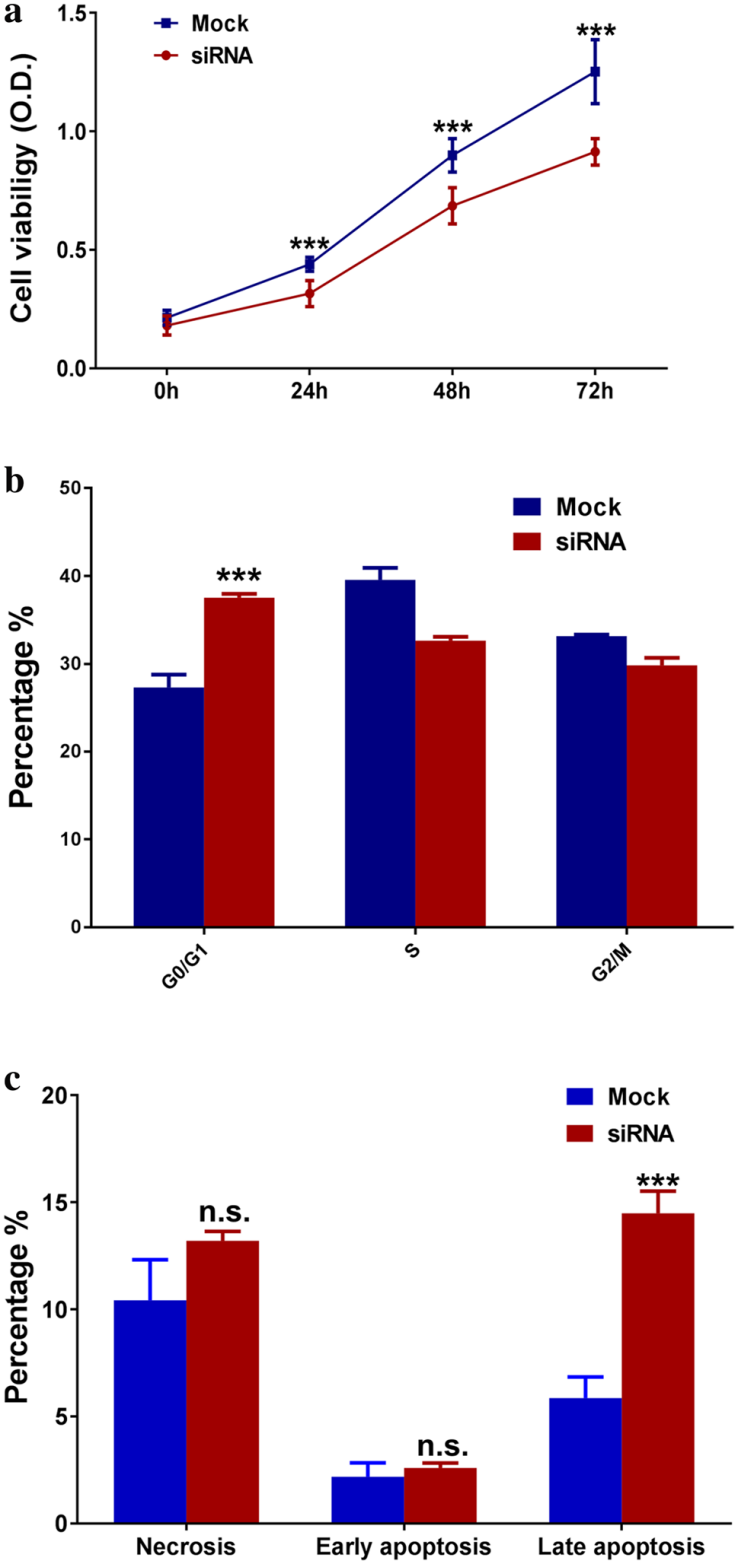
The effect of CTD-2510F5.4 knock down on cell viability, cell cycle distribution and apoptosis in HGC-27 cell line analyzed by CCK-8 assay and flow cytometry. **a** CCK-8 analysis of HGC-27 cell viability after CTD-2510F5.4 knock down. **b** Flow cytometry analysis showing cell cycle regulation of HGC-27 cells by CTD-2510F5.4 knock down. **c** Flow cytometry analysis showing percentage of apoptotic and necrotic HGC-27 cells between mock and siRNA groups. Data shown were means from three independent experiments ± SD. *** Indicates a statistical significant difference (*p* < 0.001, by Student’s *t* test)

The impact of CTD-2510F5.4 on the cell viability of gastric cancer cells was detected by the CCK-8 assay (Fig. 4a). Compared to the mock cells, CTD-2510F5.4 knock down significantly reduced the cell viability after 24 h of transfection, and such effect was constantly seen after 48 and 72 h (*p* < 0.001), suggesting CTD-2510F5.4 knock down could cause cell death in the gastric cancer cells.

### Regulation of cell cycle and apoptosis by CTD-2510F5.4 knock down

Downregulation of CTD-2510F5.4 caused reduced expression of cell cycle related genes, which prompted us to investigate if CTD-2510F5.4 was functionally related to cell cycle distribution. As shown in Fig. 4b, the percentage of cells in the G0/G1 phase increased from 27.3% (mock group) to 37.5% (CTD-2510F5.4 silencing group), suggesting induction of G0/G1 cell cycle arrest in the absence of CTD-2510F5.4 (*p* < 0.001).

The relationship between CTD-2510F5.4 and apoptosis was also investigated by flow cytometry (Fig. 4c). In results, there was a 2.5-fold change of increase in the late apoptotic cells in the CTD-2510F5.4 knock down cells (14.5%) when compared with mock cells (5.9%) (*p* < 0.001). No significant difference of necrotic cells or early apoptotic cells was observed between the two groups. These results implied the impact of CTD-2510F5.4 on the late apoptosis in gastric cancer cells.

### CTD-2510F5.4 expression in paired gastric cancer tissues and adjacent-normal gastric tissues

We then detected the expression level of CTD-2510F5.4 in 90 paired gastric cancer tissues and adjacent-normal gastric tissues by ISH. CTD-2510F5.4 expression was observed in nuclei and cytoplasm (Fig. S3a). Significantly higher expression level of CTD-2510F5.4 was detected in the gastric cancer tissues (159.6%, 119.6–174%) than in the normal tissues (98.2%, 62–132.2%) (*p* < 0.01), suggesting CTD-2510F5.4 may be a potential biomarker for gastric cancer (Fig. S3b).

### Receiver operating characteristic (ROC) curve analysis determined the cut-off value for CTD-2510F5.4 expression

**Fig. 5:**
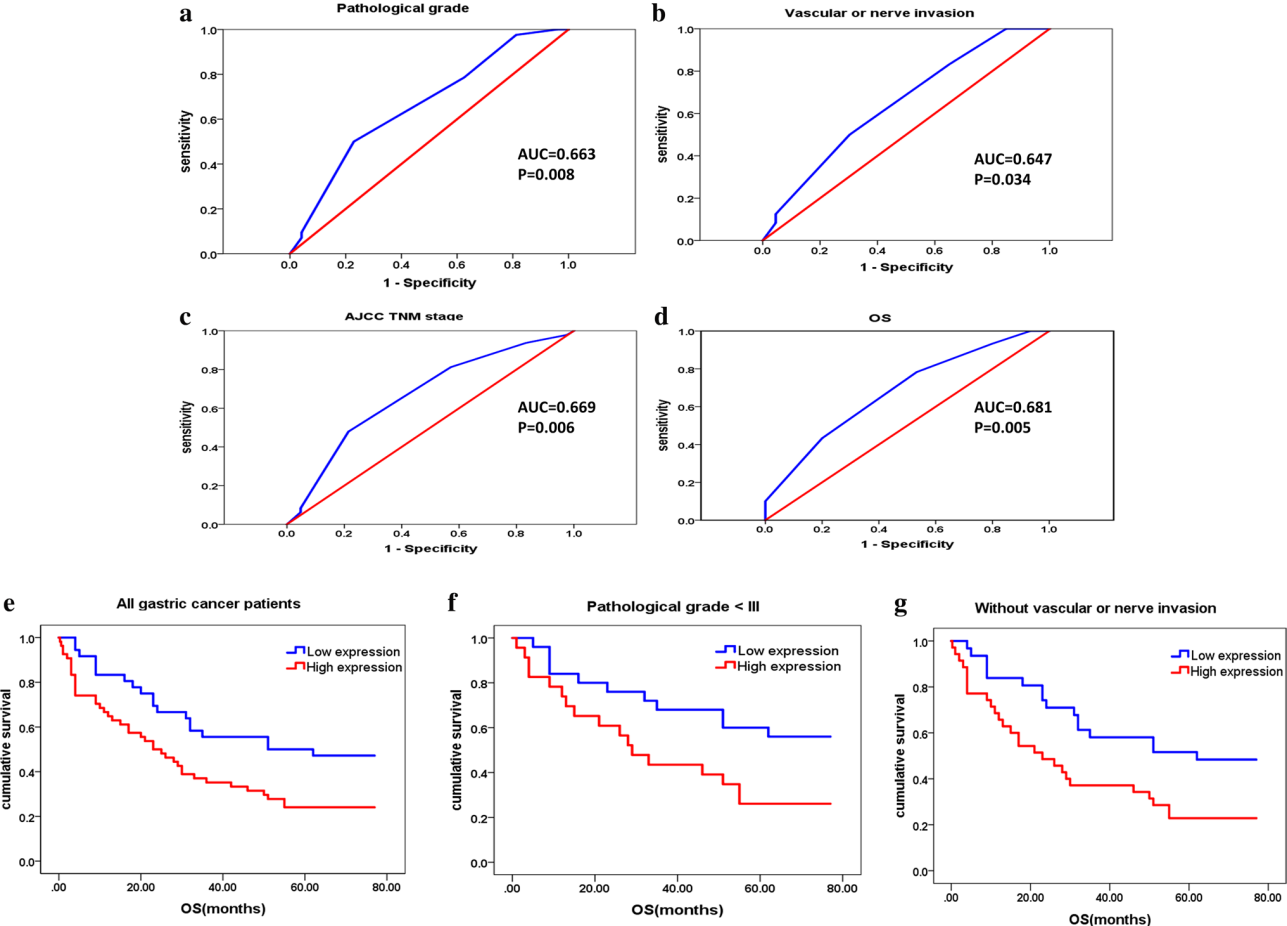
ROC curve analysis and Kaplan–Meier (K–M) analysis for CTD-2510F5.4 expression in the gastric cancers. CTD-2510F5.4 expression could be significantly distinguished by clinicopathological parameters of pathological grade (**a**), vascular or nerve invasion (**b**), AJCC TNM stage (**c**) and OS (**d**). K–M plots showed the correlations between CTD-2510F5.4 expression and the OS of **d** all gastric cancer patients, **e** gastric cancer patients at pathological grade < III, **f** gastric cancer patients without vascular or nerve invasion. K–M plots were shown for both median survival time (MST) and median OS

The clinicopathological parameters of 90 gastric cancers were listed in table S9. ROC curve analysis revealed CTD-2510F5.4 expression could be significantly distinguished by clinicopathological parameters of pathological grade, vascular or nerve invasion, AJCC TNM stage and overall survival (OS) (Fig. 5). The area under the curve (AUC) and *p* value for these parameters were 68.1% and 0.005 for OS, 64.7% and 0.034 for vascular or nerve invasion, 66.3% and 0.008 for pathological grade, and 66.9% and 0.006 for AJCC TNM stage. Cut-off value of 148.5% for CTD-2510F5.4 expression was determined, at which maximum Youden index was obtained by comparing AUC and p value for each parameter. CTD-2510F5.4 staining with H-score > 148.5% were considered as high CTD-2510F5.4 expression, and ≤ 148.5% were considered as low CTD-2510F5.4 expression. Accordingly, 36 gastric cancer tissues (40.0%) and 54 gastric cancer tissues (60.0%) showed low and high expression levels of CTD-2510F5.4, respectively.

### Correlation between CTD-2510F5.4 expression and clinicopathological parameters of gastric cancer

Having determined the cut-off value for CTD-2510F5.4 expression, we statistically analyzed the correlation between CTD-2510F5.4 expression and clinicopathological parameters of gastric cancers (Fig. 5; Table 1). (1) In regard to pathological grade, we found that high CTD-2510F5.4 expression was present in significantly more gastric cancers at pathological grade = III (73%) than those at pathological grade < III (47.9%) [adjusted *p* = 0.011, adjusted OR (95% CI) = 0.303 (0.120–0.760)]. (2) For tumor location, high CTD-2510F5.4 expression was detected in 76.2% of patients with middle third located gastric cancers, whereas 41.2% and 59.6% in patients with upper third or lower third located gastric cancers, respectively [adjusted *p* = 0.04, adjusted OR (95% CI) = 0.229 (0.056–0.935)]. (3) For depth of invasion, 68.3% of gastric tissues with serous membrane invasion and 43.3% of gastric tissues without serous membrane invasion showed high CTD-2510F5.4 expression [adjusted *p* = 0.01, adjusted OR (95% CI) = 0.271 (0.099–0.744)], respectively. (4) For vascular or nerve invasion, 79.2% of gastric tissues with vascular or nerve invasion showed high CTD-2510F5.4 expression, while 53.0% gastric tissues without vascular or nerve invasion showed high CTD-2510F5.4 expression with significant difference [adjusted *p* value = 0.028, adjusted OR (95% CI) = 0.285(0.093–0.871)]. (5) As for AJCC TNM stage, we observed more gastric cancers at III/IV stage (70.8%) obtained high CTD-2510F5.4 expression than those at I/II stage (47.6%) [adjusted *p* = 0.007, adjusted OR (95% CI) = 0.201 (0.063–0.644)]. (6) In analyzing the OS, high CTD-2510F5.4 expression was present in 68.3% deaths and 43.3% survived patients [adjusted *p* = 0.007, adjusted OR (95% CI) = 0.230 (0.078–0.673)], respectively.

**Table 1:** Correlation of CTD-2510F5.4 expression with clinicopathological parameters in patients with gastric cancer

Characteristics	CTD-2510F5.4 low expression	CTD-2510F5.4 high expression	*p* value	Adjusted OR (95%CI)
Survival status				
Survived	17 (56.7)	13 (43.3)	**0.039**	1.000
Dead	19 (31.7)	41 (68.3)	**0.007**	0.230 (0.078–0.673)
Age				
< 67	16 (40.0)	24 (60.0)	1.000	1.000
≥ 67	20 (40.0)	30 (60.0)	0.937	0.965 (0.405–2.301)
Gender				
Female	6 (27.3)	16 (72.7)	0.213	1.000
Male	30 (44.1)	38 (55.9)	0.176	2.072 (0.720–5.961)
Pathological type				
Adenocarcinoma	34 (45.3)	41 (54.7)	0.065	1.000
Mucinous adenocarcinoma	1 (20.0)	4 (80.0)	0.071	0.141 (0.017–1.182)
Signet ring cell cancer	1 (10.0)	9 (90.0)	0.535	0.380 (0.018–8.120)
Pathological grade				
< III	25 (52.1)	23 (47.9)	**0.018**	1.000
= III	11 (26.2)	31 (73.8)	**0.011**	0.303 (0.120–0.760)
Tumor size (diameter)				
< 5.5	19 (45.2)	23 (54.8)	0.392^†^	1.000
≥ 5.5	17 (35.4)	31 (64.6)	0.254^‡^	0.587 (0.235–1.467)
Tumor location				
Upper third	10 (58.8)	7 (41.2)	0.090^†^	1.000
Middle third	5 (23.8)	16 (76.2)	0.228^‡^	0.496 (0.159–1.550)
Lower third	21 (40.4)	31 (59.6)	0.189^‡^	2.186 (0.668–7.045)
Tumor type				
Early gastric cancer	2 (50.0)	2 (50.0)	0.908^†^	1.000
Borrmann type I/II/VI	10 (38.5)	16 (61.5)	0.765^‡^	0.731 (0.093–5.723)
Borrmann type III	24 (40.0)	36 (60.0)	0.879^‡^	1.081 (0.396–2.950)
Serous membrane invasion				
No	17 (56.7)	13 (43.3)	**0.039** ^†^	1.000
Yes	19 (31.7)	41 (68.3)	**0.011** ^‡^	0.271 (0.099–0.744)
Vascular or nerve invasion				
No	31 (47.0)	35 (53.0)	**0.030** ^†^	1.000
Yes	5 (20.8)	19 (79.2)	**0.028** ^‡^	0.285 (0.093–0.871)
Lymph node metastasis				
No	10 (40.0)	15 (60.0)	1.000^†^	1.000
Yes	26 (40.0)	39 (60.0)	0.887^‡^	0.932 (0.351–2.470)
AJCC TNM stage				
I/II	22 (52.4)	20 (47.6)	**0.032** ^†^	1.000
III/IV	14 (29.2)	34 (70.8)	**0.007** ^‡^	0.201 (0.063–0.644)

Bold values indicate statistical significance with *p* < 0.5

^†^*p* values were calculated by 2-sided chi-square tests or Fisher’s Exact Test

^‡^*p* values were calculated by unconditional logistic regression adjusted for gender, tumor location and lymph node metastasis

Based on these findings, we concluded that high CTD-2510F5.4 expression potentially correlated with pathological grade, tumor location, serous membrane invasion, vascular or nerve invasion and AJCC TNM stage, suggesting high CTD-2510F5.4 expression has high propensity to be present in gastric cancer patients with poor prognosis. In addition, no statistical differences were identified in regard to other parameters including age, pathological type, tumor size, tumor location, pathological morphology and lymph node metastasis.

### Correlation between CTD-2510F5.4 expression and prognosis of gastric cancer

Next, we analyzed CTD-2510F5.4 expression in all 90 cases of gastric cancers (Table 2). Median survival time (MST) was significantly shorter in cases with high CTD-2510F5.4 expression (32.849 months, 95% CI = 25.194–40.503 months) than in those with low CTD-2510F5.4 expression (49.083 months, 95% CI = 39.613–58.554 months) (*p* = 0.012, Fig. 5e). Multivariate cox analysis also revealed high CTD-2510F5.4 expression was a risk factor for the prognosis of gastric cancer patients [hazard ratio (HR, 95%CI) = 2.303 (1.316–4.028), *p* = 0.003].

**Table 2:** Association of CTD-2510F5.4 expression and clinicopathological features with OS in gastric cancer patients

Variables	OS			
	Total no.	Events no. (%)	Adjusted HR (95%CI)	*p* value
All patients				
CTD-2510F5.4 expression				
Low	36	19 (52.8)	1 (reference)	–
High	54	41 (75.9)	2.303 (1.316–4.028)	**0.003**
Pathological grade < III				
CTD-2510F5.4 expression				
Low	25	11 (44.0)	1 (reference)	–
High	23	17 (73.9)	2.362 (1.069–5.219)	**0.034**
Pathological grade = III				
CTD-2510F5.4 expression				
Low	11	8 (72.7)	1 (reference)	–
High	31	24 (77.4)	1.932 (0.827–4.517)	0.128
No vascular or nerve invasion				
CTD-2510F5.4 expression				
Low	31	16 (51.6)	1 (reference)	
High	35	27 (77.1)	2.349 (1.245–4.433)	**0.008**
With vascular or nerve invasion				
CTD-2510F5.4 expression				
Low	5	3 (60.0)	1 (reference)	
High	19	14 (73.7)	1.759 (0.481–6.433)	0.393

Bold values indicate statistical significance with *p* < 0.5

We further analyzed the correlation of CTD-2510F5.4 expression and OS stratified patient groups in regard to clinicopathological parameters. Of 48 patients with gastric cancer at pathological grade < III, there was a significant correlation between CTD-2510F5.4 expression and the patients’ OS (Fig. 5f). The MST in patients with high CTD-2510F5.4 expression (*n* = 23) was significantly shorter (37.696 months, 95% CI = 26.215–49.177 months) than in patients with low CTD-2510F5.4 expression (*n* = 25) (55.200 months, 95% CI = 44.343–66.057 months) (*p* = 0.028). Such correlation was also found in 66 patients without vascular or nerve invasion (Fig. 5g). The MST was 33.036 months and 50.742 months for patients with high CTD-2510F5.4 expression (*n* = 35) (95% CI = 23.638–42.435 months) and low CTD-2510F5.4 expression (*n* = 31) (95% CI = 40.817–60.667 months), respectively (*p* = 0.013). High CTD-2510F5.4 expression was also proved to be an independent risk factor for gastric patients with cancer at pathological grade < III (HR (95% CI) = 2.362(1.069–5.219), *p* = 0.034), or without vascular or nerve invasion [HR (95% CI) = 2.349(1.245–4.433), *p* = 0.008]. These data supported a role of high CTD-2510F5.4 expression as a risk factor in predicting poor prognosis for gastric cancers at pathological grade < III or without vascular or nerve invasion.

## Discussion

Compelling evidence has revealed the importance of lncRNAs in cancer formation and progression [14–16]. Great attentions have thereby been paid in exploring their molecular functions with the purpose of better understanding the nature of human cancers, discovering diagnostic and prognostic cancer biomarkers, and developing novel cancer therapeutic targets [17, 18]. Here we used bioinformatics approach of WGCNA to comprehensively analyze DEGs and DELs screened out between gastric cancer and adjacent normal tissues, identified co-expression network modules of hub DEGs and DELs, and ultimately found CTD-2510F5.4 was a malignant phenotype associated lncRNA with potential utility as a tissue prognostic biomarker of clinicopathological characteristics. To the best of our knowledge, this is the first time CTD-2510F5.4 was identified, and was found to be a candidate prognostic biomarker in gastric cancers.

Despite vast majority of functions remained unclear, lncRNAs have demonstrated their association with cancer phenotypes by acting as oncogenes or tumor suppressors and regulating signaling cascades. For example, Notch 1 induced LUNAR1 could drive tumor proliferation by upregulating insulin-like growth factor receptor 1 in T cell acute lymphoblastic leukemia [19]. Similarly, H19 could promote gastric cancer cell proliferation upon activated by c-myc and p53 [20, 21]. Dysregulation of HOTAIR and MALAT1 multifunctionally regulated cell cycle, apoptosis, senescence and metastasis [22, 23]. In this study, we showed that many DEGs were enriched in p53 signaling pathway, cell cycle regulation and DNA replication. When we narrowed down our investigation by constructing co-expression pathways and identified 15 hub genes, it was interesting to find again these hub genes were enriched in the process of cell replication and cell cycle regulation. Since CTD-2510F5.4 was tightly associated with 10 hub genes, implying the potential involvement of CTD-2510F5.4 in regulating cell replication and cell cycle regulation.

LncRNAs have been shown to regulate genes in *cis* manners by way of modulating neighboring intrachromosomal transcripts, or *trans* manners referring to process of epigenetically modulating distantly located transcripts. With a length of 321 nucleotides, CTD-2510F5.4 was located on chromosome 17 along with KPNA2 and KIF18B on the same chromosome, and 8 related hub genes on different chromosomes. The essential roles of these hub genes in regulating cell proliferation were well established. For example, KPNA2 and RBL1 were related to G0/G1 cell cycle transition [24, 25]. Bub1 and MCM10 were required to prevent cell cycle progression into anaphase [26], and activation of cell cycle checkpoint [27], respectively. Deficiency of E2F [28] and NUSAP1 [29] was reported to inhibit cell proliferation. Being closely related to these hub genes, CTD-2510F5.4 may also participate in cell proliferation regulatory machineries. Our study has proved the molecular function of CTD-2510F5.4 in regulating cell cycle distribution of gastric cancer cells by *in vitro* study, questions including how did CTD-2510F5.4 affect cell proliferation via these 10 hub genes were needed to be addressed in the future investigations.

Despite unidentified by bioinformatics analysis, CTD-2510F5.4 related hub genes were also likely to make contributions in the regulation of cell apoptosis. For example, deficiency of KPNA2 promoted cell apoptosis in glioblastoma multiforme [30]. Similarly, inhibition of NUSAP1 [31] or ATAD2 [32] caused apoptosis in human colorectal cancer or hepatocellular carcinoma, respectively. Therefore, silencing of CTD-2510F5.4 caused apoptosis in gastric cancer cells identified in our study may have provided preliminary evidence that CTD-2510F5.4 could regulate apoptosis via these hub genes.

Since the oncogenic and tumor suppressive roles of lncRNAs have been clarified in cancers, expectations have also been raised to determine their biomarker significance. In this context, studies exploring the prognostic and clinicopathological potentials of lncRNAs have been performed. The association between the absence of MEG3 and poor prognosis was observed in pancreatic cancers [33]. Moreover, FOXD2-AS1 served as an unfavorable prognosis biomarker in regards to poor prognosis in esophageal squamous cell carcinoma [34]. Particularly, in gastric cancer, overexpressed lncRNAs such as LINC00673 [35], LincRNAFEZF1-AS1 [36], PVT1 [37] and ANRIL [38] were found to be indicators for poor prognosis. In addition, these lncRNAs displayed oncogenic characters by mediating cancer cell behaviors including proliferation, apoptosis, migration, invasion and metastasis. Our study has unveiled the prognostic potential of CTD-2510F5.4. Using a well-characterized series of paired cancerous and normal cases, we showed significantly higher expression level of CTD-2510F5.4 in the gastric cancer tissues, implying the important role of CTD-2510F5.4 with acquisition of the malignant phenotype, again consistent with our hypothesis discussed above. Importantly, the significant association between high CTD-2510F5.4 expression with pathological grade, depth of invasion, vascular invasion, AJCC stage and OS, suggesting the potential utility of CTD-2510F5.4 as a clinical prognostic biomarker by which gastric cancer patients can be risk stratified.

Successful detection of elevated circulating lncRNAs in blood fluids encompassing urine and blood has demonstrated potential clinical application of lncRNAs as non-invasive cancer diagnostic biomarkers [39–41]. It will be of great interest to determine whether commensurate changes in the level of CTD-2510F5.4 will be detected in peripheral blood or urine in patients with gastric cancers.

In conclusion, we report that CTD-2510F5.4 was a malignant phenotype associated lncRNA, and a potential novel unfavorable prognostic biomarker for gastric cancers. The potential clinical utility of CTD-2510F5.4 in this respect remained needed to be verified in larger cohorts of gastric cancer patients.

## Electronic supplementary material

Below is the link to the electronic supplementary material.

Supplementary material 1 (PDF 2698 KB)
