# Supplementary material for: Prognostic and clinicopathological significance of long noncoding RNA CTD-2510F5.4 in gastric cancer
Source: Gastric Cancer. 2018 Dec 17;22(4):692–704. doi: 10.1007/s10120-018-00911-x (PMC6570689; doi:10.1007/s10120-018-00911-x)
Supplement: Supplementary file 1 — Supplementary material 1 (PDF 2698 KB) [file 10120_2018_911_MOESM1_ESM.pdf]

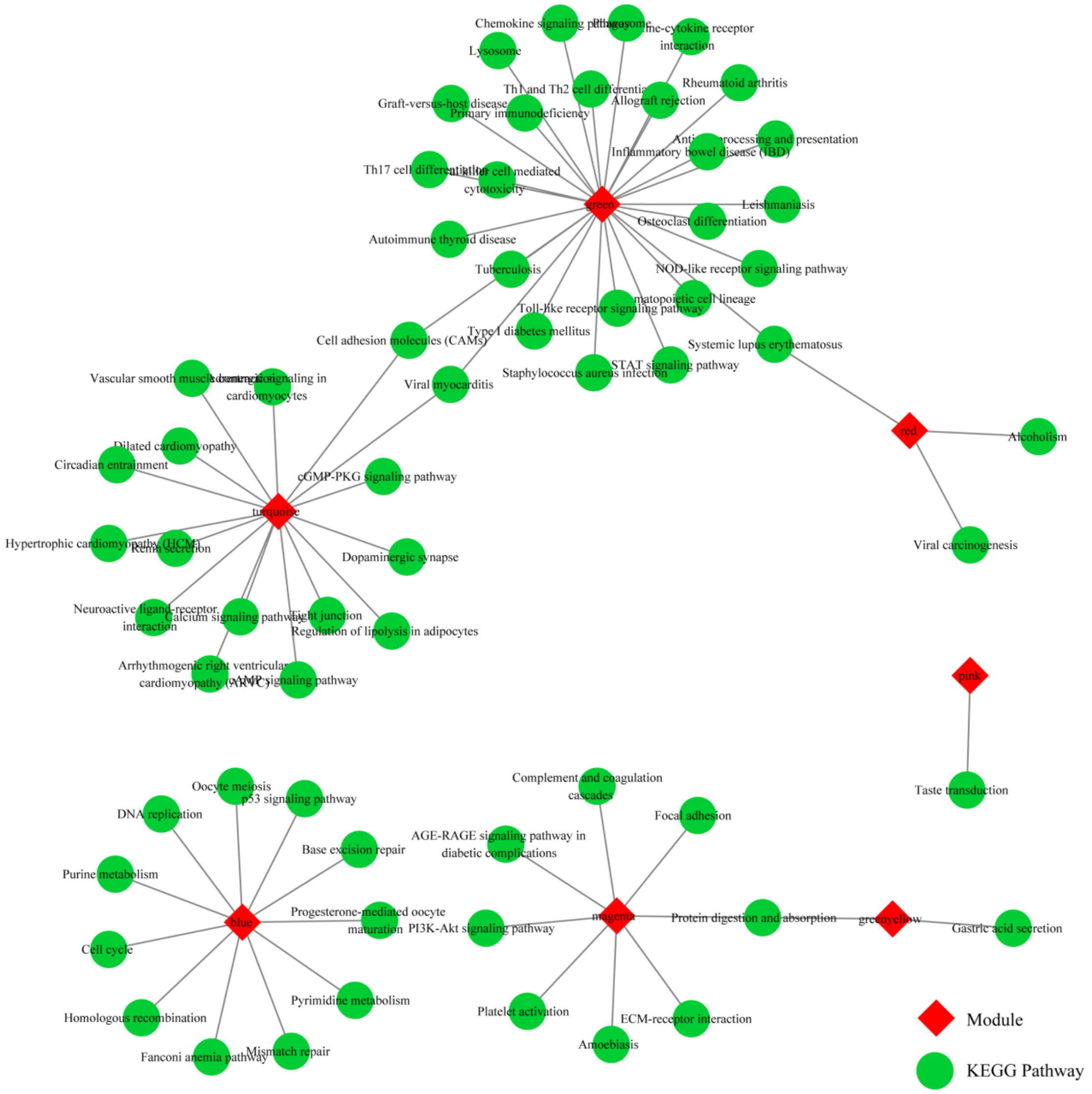

Fig. S1: KEGG pathway analysis showing 7 modules were enriched in 64 pathways, and distinct enriched pathways were displayed between different modules.

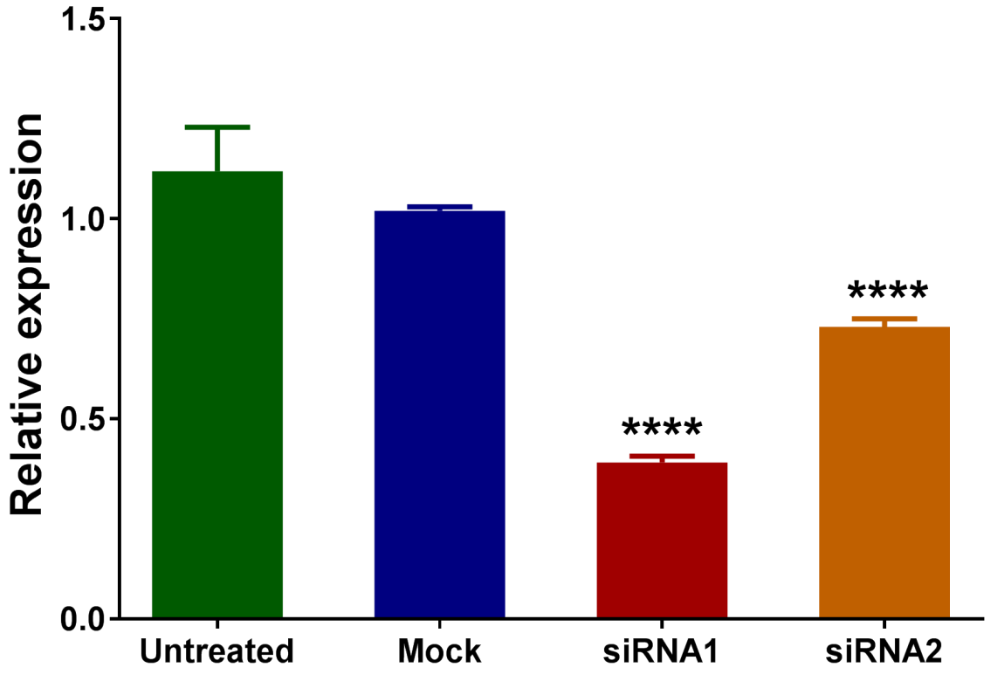

Fig. S2: Knock down of CTD-2510F5.4 by siRNA transfection in HGC-27 cell line. qRT-PCR analysis showing effective reduction in CTD-2510F5.4 gene expression after 48 hours of siRNA1 transfection (more than 60%, $p<0.0001$), and only approximately 30% of reduced gene expression by siRNA2 transfection ($p<0.0001$). Thus, siRNA1 was used in the following functional assays. The mRNA expression level of CTD-2510F5.4 was normalized against the endogenous control of GAPDH.

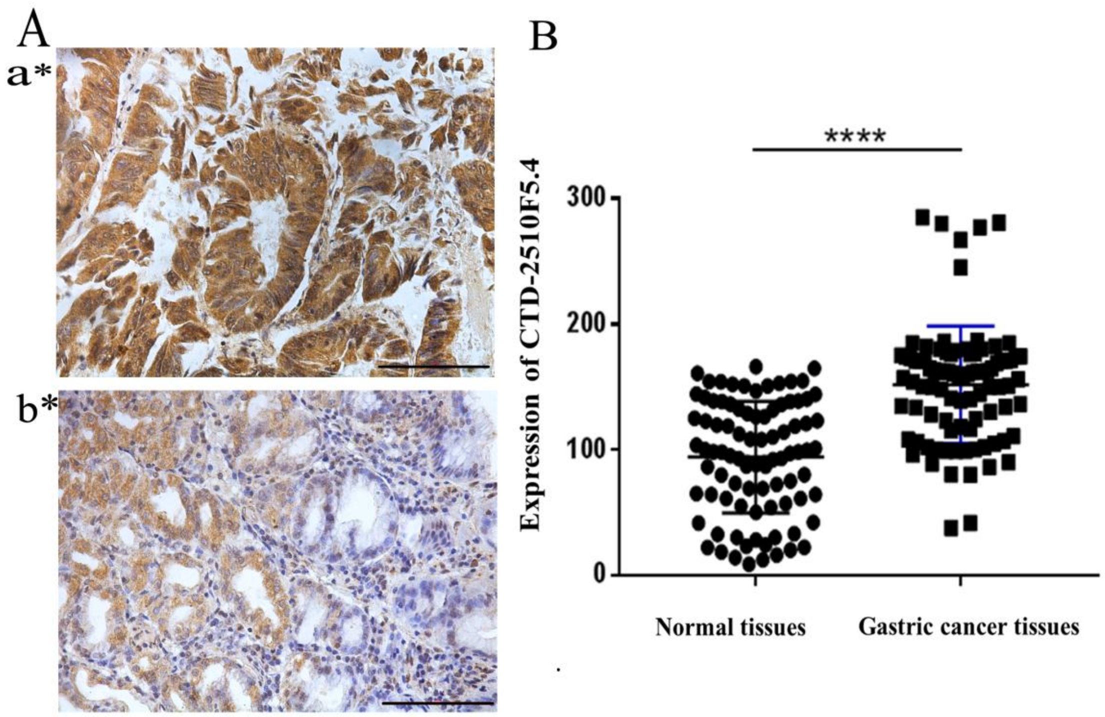

Fig. S3. CTD-2510F5.4 expression in 90 paired gastric cancer tissues and adjacent-normal gastric tissues. (a) Representative images of CTD-2510F5.4 expression detected by ISH in a gastric cancer tissue (a*) and a normal gastric tissue (b*) (20×). Scale bar=20um. (b) Comparison of CTD-2510F5.4 expression level in gastric cancer tissues and adjacent-normal gastric tissues by wilcoxon signed-rank test ($p<0.01$), data shown were mean±SD.

Table S1: KEGG analysis for DEGs.

ID	Description	GeneRatio	BgRatio	pvalue	p.adjust	qvalue	Count
hsa04110	Cell cycle	43/909	122/7249	6.43E-11	1.19E-08	1.13E-08	43
hsa03030	DNA replication	21/909	36/7249	8.12E-11	1.19E-08	1.13E-08	21
hsa03460	Fanconi anemia pathway	19/909	55/7249	2.03E-05	0.001986	0.001884	19
hsa03440	Homologous recombination	15/909	41/7249	7.08E-05	0.004586	0.004349	15
hsa04514	Cell adhesion molecules (CAMs)	35/909	145/7249	7.83E-05	0.004586	0.004349	35
hsa04020	Calcium signaling pathway	41/909	182/7249	0.000106	0.00518	0.004913	41
hsa04080	Neuroactive ligand-receptor interaction	55/909	278/7249	0.000313	0.013111	0.012435	55
hsa04974	Protein digestion and absorption	23/909	90/7249	0.000544	0.019913	0.018887	23
hsa05322	Systemic lupus erythematosus	30/909	133/7249	0.000847	0.027558	0.026138	30
hsa04115	p53 signaling pathway	18/909	67/7249	0.001139	0.03338	0.031659	18
hsa05323	Rheumatoid arthritis	22/909	90/7249	0.001348	0.035898	0.034047	22
hsa00240	Pyrimidine metabolism	24/909	102/7249	0.001471	0.03591	0.034059	24

Table S2: Biological process of GO analysis on DEGs.

ID	Description	Gene Ratio	Bg Ratio	p value	p.adjust	q value	Count
GO:0007059	chromosome segregation	112/2102	330/16992	7.10E-25	3.51E-21	2.86E-21	112
GO:0007067	mitotic nuclear division	137/2102	453/16992	1.25E-24	3.51E-21	2.86E-21	137
GO:0098813	nuclear chromosome segregation	99/2102	287/16992	1.03E-22	1.93E-19	1.57E-19	99
GO:0000819	sister chromatid segregation	83/2102	218/16992	2.06E-22	2.90E-19	2.36E-19	83
GO:0006260	DNA replication	94/2102	291/16992	2.19E-19	2.47E-16	2.01E-16	94
GO:0006261	DNA-dependent DNA replication	59/2102	138/16992	3.54E-19	3.32E-16	2.70E-16	59
GO:0006270	DNA replication initiation	26/2102	39/16992	3.51E-15	2.83E-12	2.30E-12	26
GO:0000070	mitotic sister chromatid segregation	51/2102	134/16992	2.72E-14	1.92E-11	1.56E-11	51
GO:0007062	sister chromatid cohesion	49/2102	126/16992	3.27E-14	2.05E-11	1.67E-11	49
GO:0000082	G1/S transition of mitotic cell cycle	69/2102	241/16992	8.69E-12	4.61E-09	3.75E-09	69
GO:0071103	DNA conformation change	77/2102	283/16992	9.00E-12	4.61E-09	3.75E-09	77
GO:0003012	muscle system process	98/2102	402/16992	1.63E-11	7.65E-09	6.22E-09	98
GO:0044843	cell cycle G1/S phase transition	70/2102	254/16992	4.21E-11	1.83E-08	1.49E-08	70
GO:0006936	muscle contraction	84/2102	331/16992	5.34E-11	2.15E-08	1.75E-08	84
GO:0051321	meiotic cell cycle	65/2102	233/16992	1.18E-10	4.45E-08	3.62E-08	65
GO:0006310	DNA recombination	71/2102	267/16992	1.79E-10	6.31E-08	5.13E-08	71
GO:0007052	mitotic spindle organization	35/2102	91/16992	2.13E-10	6.66E-08	5.42E-08	35
GO:1902850	microtubule cytoskeleton organization involved in mitosis	35/2102	91/16992	2.13E-10	6.66E-08	5.42E-08	35
GO:0007051	spindle organization	47/2102	146/16992	2.52E-10	7.47E-08	6.08E-08	47
GO:0000226	microtubule cytoskeleton organization	100/2102	443/16992	1.01E-09	2.85E-07	2.32E-07	100
GO:0051983	regulation of chromosome segregation	32/2102	83/16992	1.17E-09	3.14E-07	2.55E-07	32
GO:0030198	extracellular matrix organization	77/2102	315/16992	2.15E-09	5.51E-07	4.48E-07	77
GO:0043062	extracellular structure organization	77/2102	316/16992	2.50E-09	6.14E-07	4.99E-07	77
GO:0006323	DNA packaging	55/2102	199/16992	4.51E-09	1.06E-06	8.61E-07	55
GO:0000075	cell cycle checkpoint	59/2102	221/16992	5.22E-09	1.18E-06	9.56E-07	59
GO:1903046	meiotic cell cycle process	54/2102	195/16992	5.77E-09	1.25E-06	1.02E-06	54
GO:0032963	collagen metabolic process	37/2102	111/16992	6.87E-09	1.43E-06	1.17E-06	37
GO:0044236	multicellular organism metabolic process	42/2102	136/16992	8.96E-09	1.80E-06	1.47E-06	42
GO:0007126	meiotic nuclear division	51/2102	184/16992	1.47E-08	2.85E-06	2.32E-06	51
GO:0008016	regulation of heart	59/2102	229/16992	2.15E-08	3.88E-06	3.16E-06	59

	contraction						
GO:0071897	DNA biosynthetic process	54/2102	202/16992	2.19E-08	3.88E-06	3.16E-06	54
GO:0044057	regulation of system process	104/2102	493/16992	2.21E-08	3.88E-06	3.16E-06	104
GO:0044259	multicellular organismal macromolecule metabolic process	37/2102	116/16992	2.61E-08	4.44E-06	3.61E-06	37
GO:0001655	urogenital system development	74/2102	315/16992	2.68E-08	4.44E-06	3.61E-06	74
GO:0007088	regulation of mitotic nuclear division	44/2102	151/16992	2.82E-08	4.54E-06	3.69E-06	44
GO:0008015	blood circulation	104/2102	496/16992	3.06E-08	4.79E-06	3.90E-06	104
GO:0033260	nuclear DNA replication	16/2102	29/16992	3.94E-08	6.01E-06	4.89E-06	16
GO:0003013	circulatory system process	104/2102	500/16992	4.71E-08	6.98E-06	5.68E-06	104
GO:0042391	regulation of membrane potential	84/2102	379/16992	5.28E-08	7.63E-06	6.20E-06	84
GO:0072001	renal system development	67/2102	280/16992	5.71E-08	8.05E-06	6.55E-06	67
GO:0006312	mitotic recombination	21/2102	49/16992	9.67E-08	1.33E-05	1.08E-05	21
GO:0051783	regulation of nuclear division	47/2102	173/16992	1.04E-07	1.39E-05	1.13E-05	47
GO:2000021	regulation of ion homeostasis	51/2102	195/16992	1.14E-07	1.50E-05	1.22E-05	51
GO:0044786	cell cycle DNA replication	18/2102	38/16992	1.23E-07	1.58E-05	1.28E-05	18
GO:0001501	skeletal system development	100/2102	485/16992	1.33E-07	1.67E-05	1.36E-05	100
GO:0032844	regulation of homeostatic process	94/2102	449/16992	1.52E-07	1.86E-05	1.52E-05	94
GO:0001822	kidney development	63/2102	265/16992	1.81E-07	2.17E-05	1.77E-05	63
GO:0030574	collagen catabolic process	25/2102	68/16992	2.23E-07	2.56E-05	2.09E-05	25
GO:0051304	chromosome separation	25/2102	68/16992	2.23E-07	2.56E-05	2.09E-05	25
GO:0006271	DNA strand elongation involved in DNA replication	11/2102	16/16992	2.44E-07	2.69E-05	2.19E-05	11
GO:0034501	protein localization to kinetochore	11/2102	16/16992	2.44E-07	2.69E-05	2.19E-05	11
GO:1903522	regulation of blood circulation	64/2102	273/16992	2.49E-07	2.70E-05	2.19E-05	64
GO:0071459	protein localization to chromosome, centromeric region	12/2102	19/16992	2.71E-07	2.88E-05	2.35E-05	12
GO:0051052	regulation of DNA metabolic process	81/2102	376/16992	3.21E-07	3.35E-05	2.72E-05	81
GO:0008608	attachment of spindle microtubules to kinetochore	15/2102	29/16992	3.27E-07	3.35E-05	2.72E-05	15
GO:0070507	regulation of microtubule cytoskeleton organization	38/2102	132/16992	3.43E-07	3.45E-05	2.81E-05	38
GO:0034502	protein localization to	25/2102	70/16992	4.24E-07	4.13E-05	3.36E-05	25

	chromosome						
GO:0000732	strand displacement	14/2102	26/16992	4.25E-07	4.13E-05	3.36E-05	14
GO:0035637	multicellular organismal signaling	49/2102	192/16992	4.47E-07	4.23E-05	3.44E-05	49
GO:0048660	regulation of smooth muscle cell proliferation	34/2102	113/16992	4.51E-07	4.23E-05	3.44E-05	34
GO:0033002	muscle cell proliferation	44/2102	166/16992	5.54E-07	5.12E-05	4.17E-05	44
GO:0003015	heart process	61/2102	262/16992	6.07E-07	5.46E-05	4.44E-05	61
GO:0090257	regulation of muscle system process	51/2102	205/16992	6.10E-07	5.46E-05	4.44E-05	51
GO:0043270	positive regulation of ion transport	54/2102	222/16992	6.22E-07	5.48E-05	4.45E-05	54
GO:0034508	centromere complex assembly	21/2102	54/16992	6.95E-07	6.03E-05	4.90E-05	21
GO:0031055	chromatin remodeling at centromere	19/2102	46/16992	7.86E-07	6.59E-05	5.36E-05	19
GO:0034080	CENP-A containing nucleosome assembly	18/2102	42/16992	7.95E-07	6.59E-05	5.36E-05	18
GO:0061641	CENP-A containing chromatin organization	18/2102	42/16992	7.95E-07	6.59E-05	5.36E-05	18
GO:0048659	smooth muscle cell proliferation	34/2102	116/16992	8.86E-07	7.20E-05	5.85E-05	34
GO:0060047	heart contraction	60/2102	259/16992	8.94E-07	7.20E-05	5.85E-05	60
GO:0006937	regulation of muscle contraction	40/2102	148/16992	1.04E-06	8.27E-05	6.73E-05	40
GO:0030199	collagen fibril organization	17/2102	39/16992	1.20E-06	9.43E-05	7.67E-05	17
GO:0090068	positive regulation of cell cycle process	58/2102	250/16992	1.28E-06	9.92E-05	8.07E-05	58
GO:0072006	nephron development	38/2102	139/16992	1.42E-06	0.000108	8.77E-05	38
GO:0000281	mitotic cytokinesis	16/2102	36/16992	1.81E-06	0.000134	0.000109	16
GO:0031577	spindle checkpoint	16/2102	36/16992	1.81E-06	0.000134	0.000109	16
GO:0043486	histone exchange	20/2102	53/16992	2.23E-06	0.000163	0.000132	20
GO:0044243	multicellular organismal catabolic process	25/2102	76/16992	2.45E-06	0.000177	0.000144	25
GO:0007100	mitotic centrosome separation	9/2102	13/16992	2.99E-06	0.000213	0.000173	9
GO:0045787	positive regulation of cell cycle	72/2102	341/16992	3.09E-06	0.000217	0.000177	72
GO:0010959	regulation of metal ion transport	69/2102	323/16992	3.21E-06	0.000224	0.000182	69
GO:0060402	calcium ion transport into cytosol	35/2102	128/16992	3.61E-06	0.000248	0.000202	35
GO:0061640	cytoskeleton-dependent cytokinesis	18/2102	46/16992	3.88E-06	0.000262	0.000213	18
GO:0000731	DNA synthesis involved in DNA repair	24/2102	73/16992	3.91E-06	0.000262	0.000213	24
GO:0010522	regulation of calcium ion transport into cytosol	27/2102	88/16992	4.44E-06	0.000293	0.000238	27
GO:0051928	positive regulation of calcium ion transport	29/2102	98/16992	4.47E-06	0.000293	0.000238	29
GO:0007093	mitotic cell cycle checkpoint	39/2102	151/16992	4.73E-06	0.000307	0.000249	39

GO:0000083	regulation of transcription involved in G1/S transition of mitotic cell cycle	13/2102	27/16992	5.69E-06	0.000364	0.000296	13
GO:0006302	double-strand break repair	49/2102	209/16992	6.11E-06	0.000387	0.000315	49
GO:0007271	synaptic transmission, cholinergic	15/2102	35/16992	6.61E-06	0.000414	0.000337	15
GO:0090279	regulation of calcium ion import	29/2102	100/16992	6.94E-06	0.00043	0.00035	29
GO:0051054	positive regulation of DNA metabolic process	49/2102	210/16992	7.03E-06	0.000431	0.00035	49
GO:0061337	cardiac conduction	36/2102	137/16992	7.11E-06	0.000431	0.000351	36
GO:0070509	calcium ion import	40/2102	159/16992	7.19E-06	0.000431	0.000351	40
GO:0051299	centrosome separation	9/2102	14/16992	7.44E-06	0.000442	0.000359	9
GO:0090329	regulation of DNA-dependent DNA replication	17/2102	44/16992	8.80E-06	0.000517	0.00042	17
GO:0032886	regulation of microtubule-based process	39/2102	155/16992	9.27E-06	0.000536	0.000436	39
GO:0006334	nucleosome assembly	37/2102	144/16992	9.33E-06	0.000536	0.000436	37
GO:0006336	DNA replication-independent nucleosome assembly	19/2102	53/16992	9.55E-06	0.000544	0.000442	19
GO:0031497	chromatin assembly	40/2102	161/16992	9.93E-06	0.00056	0.000455	40
GO:0000722	telomere maintenance via recombination	15/2102	36/16992	1.00E-05	0.00056	0.000456	15
GO:0007098	centrosome cycle	25/2102	82/16992	1.12E-05	0.000611	0.000497	25
GO:0045132	meiotic chromosome segregation	26/2102	87/16992	1.13E-05	0.000611	0.000497	26
GO:0051225	spindle assembly	26/2102	87/16992	1.13E-05	0.000611	0.000497	26
GO:1901990	regulation of mitotic cell cycle phase transition	66/2102	316/16992	1.14E-05	0.000611	0.000497	66
GO:0051297	centrosome organization	31/2102	113/16992	1.19E-05	0.00063	0.000513	31
GO:0032835	glomerulus development	21/2102	63/16992	1.22E-05	0.000642	0.000522	21
GO:0034724	DNA replication-independent nucleosome organization	19/2102	54/16992	1.30E-05	0.00068	0.000553	19
GO:0007080	mitotic metaphase plate congression	16/2102	41/16992	1.39E-05	0.000717	0.000583	16
GO:0051383	kinetochore organization	10/2102	18/16992	1.40E-05	0.000717	0.000583	10
GO:0071173	spindle assembly checkpoint	13/2102	29/16992	1.51E-05	0.000769	0.000625	13
GO:0033045	regulation of sister chromatid segregation	21/2102	64/16992	1.60E-05	0.000807	0.000656	21
GO:0006275	regulation of DNA replication	31/2102	115/16992	1.74E-05	0.000855	0.000695	31
GO:0051924	regulation of calcium ion transport	47/2102	205/16992	1.75E-05	0.000855	0.000695	47
GO:0007091	metaphase/anaphase transition of mitotic	17/2102	46/16992	1.76E-05	0.000855	0.000695	17

	cell cycle						
GO:0010965	regulation of mitotic sister chromatid separation	17/2102	46/16992	1.76E-05	0.000855	0.000695	17
GO:0007517	muscle organ development	71/2102	353/16992	2.06E-05	0.000992	0.000807	71
GO:0061448	connective tissue development	52/2102	236/16992	2.08E-05	0.000994	0.000809	52
GO:1903779	regulation of cardiac conduction	22/2102	70/16992	2.19E-05	0.001038	0.000844	22
GO:0030261	chromosome condensation	14/2102	34/16992	2.33E-05	0.001095	0.000891	14
GO:0031570	DNA integrity checkpoint	39/2102	161/16992	2.38E-05	0.001109	0.000902	39
GO:0043113	receptor clustering	17/2102	47/16992	2.44E-05	0.001109	0.000902	17
GO:0044784	metaphase/anaphase transition of cell cycle	17/2102	47/16992	2.44E-05	0.001109	0.000902	17
GO:1905818	regulation of chromosome separation	17/2102	47/16992	2.44E-05	0.001109	0.000902	17
GO:0007568	aging	59/2102	280/16992	2.48E-05	0.001117	0.000908	59
GO:0000724	double-strand break repair via homologous recombination	28/2102	101/16992	2.50E-05	0.001117	0.000909	28
GO:0060401	cytosolic calcium ion transport	35/2102	139/16992	2.56E-05	0.001138	0.000925	35
GO:0031099	regeneration	41/2102	173/16992	2.59E-05	0.001142	0.000929	41
GO:0048568	embryonic organ development	81/2102	420/16992	2.75E-05	0.001203	0.000979	81
GO:0051279	regulation of release of sequestered calcium ion into cytosol	22/2102	71/16992	2.81E-05	0.001217	0.00099	22
GO:0007127	meiosis I	29/2102	107/16992	2.87E-05	0.001226	0.000997	29
GO:0051208	sequestering of calcium ion	29/2102	107/16992	2.87E-05	0.001226	0.000997	29
GO:0000725	recombinational repair	28/2102	102/16992	3.04E-05	0.001289	0.001048	28
GO:0022616	DNA strand elongation	11/2102	23/16992	3.28E-05	0.001381	0.001123	11
GO:0046605	regulation of centrosome cycle	17/2102	48/16992	3.35E-05	0.001386	0.001127	17
GO:0051306	mitotic sister chromatid separation	17/2102	48/16992	3.35E-05	0.001386	0.001127	17
GO:1904385	cellular response to angiotensin	9/2102	16/16992	3.37E-05	0.001386	0.001127	9
GO:0098801	regulation of renal system process	14/2102	35/16992	3.45E-05	0.001408	0.001145	14
GO:0031023	microtubule organizing center organization	31/2102	119/16992	3.60E-05	0.00146	0.001187	31
GO:0051209	release of sequestered calcium ion into cytosol	28/2102	103/16992	3.69E-05	0.001475	0.0012	28
GO:0051283	negative regulation of sequestering of calcium ion	28/2102	103/16992	3.69E-05	0.001475	0.0012	28
GO:1902969	mitotic DNA replication	7/2102	10/16992	3.75E-05	0.001487	0.001209	7
GO:0090307	mitotic spindle assembly	18/2102	53/16992	3.79E-05	0.001493	0.001214	18

GO:0030071	regulation of mitotic metaphase/anaphase transition	16/2102	44/16992	3.90E-05	0.001528	0.001243	16
GO:0051216	cartilage development	42/2102	182/16992	4.07E-05	0.001581	0.001286	42
GO:0006941	striated muscle contraction	39/2102	165/16992	4.30E-05	0.00166	0.00135	39
GO:0034728	nucleosome organization	40/2102	171/16992	4.44E-05	0.001699	0.001382	40
GO:0051282	regulation of sequestering of calcium ion	28/2102	104/16992	4.46E-05	0.001699	0.001382	28
GO:0045930	negative regulation of mitotic cell cycle	57/2102	274/16992	4.93E-05	0.001864	0.001516	57
GO:1902099	regulation of metaphase/anaphase transition of cell cycle	16/2102	45/16992	5.37E-05	0.002017	0.00164	16
GO:0051310	metaphase plate congression	17/2102	50/16992	6.09E-05	0.002273	0.001848	17
GO:0000910	cytokinesis	34/2102	139/16992	6.26E-05	0.002322	0.001888	34
GO:0045931	positive regulation of mitotic cell cycle	32/2102	128/16992	6.49E-05	0.002391	0.001944	32
GO:0008217	regulation of blood pressure	39/2102	168/16992	6.56E-05	0.002402	0.001954	39
GO:0006942	regulation of striated muscle contraction	24/2102	85/16992	6.72E-05	0.002443	0.001987	24
GO:0006333	chromatin assembly or disassembly	42/2102	186/16992	6.93E-05	0.002504	0.002036	42
GO:0048662	negative regulation of smooth muscle cell proliferation	14/2102	37/16992	7.13E-05	0.002561	0.002083	14
GO:1901654	response to ketone	41/2102	181/16992	7.80E-05	0.002782	0.002263	41
GO:0090280	positive regulation of calcium ion import	17/2102	51/16992	8.09E-05	0.002866	0.002331	17
GO:0007076	mitotic chromosome condensation	8/2102	14/16992	8.13E-05	0.002866	0.002331	8
GO:0002824	positive regulation of adaptive immune response based on somatic recombination of immune receptors built from immunoglobulin superfamily domains	22/2102	76/16992	8.87E-05	0.003104	0.002524	22
GO:0006939	smooth muscle contraction	26/2102	97/16992	8.92E-05	0.003104	0.002524	26
GO:1901987	regulation of cell cycle phase transition	66/2102	337/16992	9.20E-05	0.003161	0.002571	66
GO:0051988	regulation of attachment of spindle microtubules to kinetochore	7/2102	11/16992	9.20E-05	0.003161	0.002571	7
GO:0006338	chromatin remodeling	37/2102	159/16992	9.45E-05	0.003228	0.002625	37
GO:0060993	kidney morphogenesis	25/2102	92/16992	9.51E-05	0.003228	0.002625	25
GO:0032845	negative regulation of homeostatic process	41/2102	183/16992	0.000101	0.00341	0.002773	41
GO:0097553	calcium ion transmembrane import into cytosol	28/2102	109/16992	0.00011	0.003654	0.002971	28

GO:1902656	calcium ion import into cytosol	28/2102	109/16992	0.00011	0.003654	0.002971	28
GO:0048732	gland development	78/2102	417/16992	0.000111	0.003659	0.002975	78
GO:0006813	potassium ion transport	48/2102	226/16992	0.000111	0.003659	0.002975	48
GO:0048557	embryonic digestive tract morphogenesis	9/2102	18/16992	0.000113	0.003685	0.002997	9
GO:1990776	response to angiotensin	9/2102	18/16992	0.000113	0.003685	0.002997	9
GO:0048566	embryonic digestive tract development	13/2102	34/16992	0.000114	0.003685	0.002997	13
GO:0007204	positive regulation of cytosolic calcium ion concentration	54/2102	264/16992	0.000122	0.003935	0.0032	54
GO:0010035	response to inorganic substance	88/2102	485/16992	0.000127	0.004058	0.0033	88
GO:1904062	regulation of cation transmembrane transport	52/2102	252/16992	0.000128	0.004058	0.0033	52
GO:0002825	regulation of T-helper 1 type immune response	10/2102	22/16992	0.000129	0.004058	0.0033	10
GO:0036297	interstrand cross-link repair	16/2102	48/16992	0.00013	0.004058	0.0033	16
GO:0071804	cellular potassium ion transport	42/2102	191/16992	0.00013	0.004058	0.0033	42
GO:0071805	potassium ion transmembrane transport	42/2102	191/16992	0.00013	0.004058	0.0033	42
GO:0060706	cell differentiation involved in embryonic placenta development	11/2102	26/16992	0.000132	0.004076	0.003315	11
GO:0002287	alpha-beta T cell activation involved in immune response	17/2102	53/16992	0.000139	0.00423	0.00344	17
GO:0002293	alpha-beta T cell differentiation involved in immune response	17/2102	53/16992	0.000139	0.00423	0.00344	17
GO:0033047	regulation of mitotic sister chromatid segregation	17/2102	53/16992	0.000139	0.00423	0.00344	17
GO:0032200	telomere organization	37/2102	162/16992	0.000142	0.004315	0.003509	37
GO:2000573	positive regulation of DNA biosynthetic process	19/2102	63/16992	0.000145	0.004371	0.003555	19
GO:0042692	muscle cell differentiation	69/2102	362/16992	0.000152	0.004543	0.003694	69
GO:0032392	DNA geometric change	23/2102	84/16992	0.000157	0.004695	0.003818	23
GO:0021537	telencephalon development	49/2102	236/16992	0.000169	0.005022	0.004084	49
GO:0072073	kidney epithelium development	33/2102	140/16992	0.00017	0.005026	0.004087	33
GO:0050771	negative regulation of axonogenesis	16/2102	49/16992	0.000171	0.00503	0.004091	16
GO:0032508	DNA duplex unwinding	21/2102	74/16992	0.000173	0.005039	0.004098	21
GO:0043044	ATP-dependent chromatin remodeling	21/2102	74/16992	0.000173	0.005039	0.004098	21
GO:0007178	transmembrane receptor protein serine/threonine kinase	61/2102	312/16992	0.000175	0.005052	0.004109	61

	signaling pathway						
GO:0060688	regulation of morphogenesis of a branching structure	17/2102	54/16992	0.000179	0.005137	0.004178	17
GO:0060537	muscle tissue development	67/2102	351/16992	0.00018	0.005137	0.004178	67
GO:0003014	renal system process	28/2102	112/16992	0.000181	0.005137	0.004178	28
GO:0045005	DNA-dependent DNA replication maintenance of fidelity	12/2102	31/16992	0.000181	0.005137	0.004178	12
GO:0002292	T cell differentiation involved in immune response	18/2102	59/16992	0.000183	0.005157	0.004194	18
GO:0051302	regulation of cell division	32/2102	135/16992	0.00019	0.005331	0.004335	32
GO:0003093	regulation of glomerular filtration	7/2102	12/16992	0.000197	0.005481	0.004457	7
GO:0007094	mitotic spindle assembly checkpoint	11/2102	27/16992	0.000197	0.005481	0.004457	11
GO:0042472	inner ear morphogenesis	25/2102	96/16992	0.000199	0.005487	0.004462	25
GO:0002821	positive regulation of adaptive immune response	22/2102	80/16992	0.000203	0.00557	0.00453	22
GO:0001889	liver development	31/2102	130/16992	0.000212	0.005799	0.004716	31
GO:0001823	mesonephros development	26/2102	102/16992	0.000217	0.005882	0.004784	26
GO:1904064	positive regulation of cation transmembrane transport	26/2102	102/16992	0.000217	0.005882	0.004784	26
GO:0090183	regulation of kidney development	17/2102	55/16992	0.00023	0.006207	0.005048	17
GO:0003018	vascular process in circulatory system	35/2102	154/16992	0.000235	0.006302	0.005125	35
GO:0051238	sequestering of metal ion	30/2102	125/16992	0.000236	0.006302	0.005125	30
GO:0000086	G2/M transition of mitotic cell cycle	48/2102	233/16992	0.000238	0.006322	0.005142	48
GO:0030879	mammary gland development	31/2102	131/16992	0.000245	0.006491	0.005278	31
GO:0048608	reproductive structure development	77/2102	421/16992	0.000254	0.006679	0.005432	77
GO:0000723	telomere maintenance	34/2102	149/16992	0.000264	0.006934	0.005639	34
GO:0032355	response to estradiol	30/2102	126/16992	0.000274	0.007139	0.005806	30
GO:0035850	epithelial cell differentiation involved in kidney development	15/2102	46/16992	0.000276	0.007176	0.005836	15
GO:0007548	sex differentiation	53/2102	266/16992	0.000279	0.007213	0.005866	53
GO:0072507	divalent inorganic cation homeostasis	79/2102	436/16992	0.000287	0.007391	0.00601	79
GO:0051784	negative regulation of nuclear division	16/2102	51/16992	0.000288	0.007391	0.00601	16
GO:0014910	regulation of smooth muscle cell migration	17/2102	56/16992	0.000293	0.007435	0.006047	17
GO:0061005	cell differentiation involved in kidney development	17/2102	56/16992	0.000293	0.007435	0.006047	17
GO:2000147	positive regulation of	75/2102	410/16992	0.000302	0.007622	0.006198	75

	cell motility						
GO:0044839	cell cycle G2/M phase transition	49/2102	242/16992	0.000315	0.007918	0.006439	49
GO:0031100	animal organ regeneration	21/2102	77/16992	0.000317	0.007918	0.006439	21
GO:0061458	reproductive system development	77/2102	424/16992	0.000317	0.007918	0.006439	77
GO:0051101	regulation of DNA binding	27/2102	110/16992	0.000322	0.00796	0.006474	27
GO:0072009	nephron epithelium development	27/2102	110/16992	0.000322	0.00796	0.006474	27
GO:0050880	regulation of blood vessel size	31/2102	133/16992	0.000326	0.007976	0.006486	31
GO:0061008	hepaticobiliary system development	31/2102	133/16992	0.000326	0.007976	0.006486	31
GO:0043583	ear development	43/2102	205/16992	0.000327	0.007976	0.006486	43
GO:0042471	ear morphogenesis	28/2102	116/16992	0.000339	0.008233	0.006696	28
GO:0072511	divalent inorganic cation transport	78/2102	432/16992	0.00035	0.008462	0.006881	78
GO:0006816	calcium ion transport	70/2102	379/16992	0.000355	0.008556	0.006958	70
GO:0010524	positive regulation of calcium ion transport into cytosol	15/2102	47/16992	0.00036	0.008631	0.007019	15
GO:0002294	CD4-positive, alpha-beta T cell differentiation involved in immune response	16/2102	52/16992	0.000369	0.008822	0.007174	16
GO:0035150	regulation of tube size	31/2102	134/16992	0.000375	0.00892	0.007254	31
GO:0051098	regulation of binding	61/2102	321/16992	0.000386	0.00912	0.007417	61
GO:0051272	positive regulation of cellular component movement	76/2102	420/16992	0.000387	0.00912	0.007417	76
GO:0034765	regulation of ion transmembrane transport	74/2102	407/16992	0.000397	0.009318	0.007578	74
GO:0030900	forebrain development	67/2102	361/16992	0.000409	0.009483	0.007712	67
GO:0043370	regulation of CD4-positive, alpha-beta T cell differentiation	13/2102	38/16992	0.000412	0.009483	0.007712	13
GO:0003382	epithelial cell morphogenesis	11/2102	29/16992	0.000414	0.009483	0.007712	11
GO:0014808	release of sequestered calcium ion into cytosol by sarcoplasmic reticulum	11/2102	29/16992	0.000414	0.009483	0.007712	11
GO:0071174	mitotic spindle checkpoint	11/2102	29/16992	0.000414	0.009483	0.007712	11
GO:2000516	positive regulation of CD4-positive, alpha-beta T cell activation	11/2102	29/16992	0.000414	0.009483	0.007712	11
GO:0070838	divalent metal ion transport	77/2102	428/16992	0.000425	0.009704	0.007892	77
GO:0016049	cell growth	85/2102	482/16992	0.000427	0.009715	0.0079	85
GO:2000278	regulation of DNA biosynthetic process	24/2102	95/16992	0.00043	0.009738	0.007919	24

GO:0065004	protein-DNA complex assembly	48/2102	239/16992	0.000438	0.009834	0.007997	48
GO:0030335	positive regulation of cell migration	72/2102	395/16992	0.000438	0.009834	0.007997	72

Table S3: Cellular component of GO analysis on DEGs.

ID	Description	Gene Ratio	Bg Ratio	p value	p.adjust	q value	Count
GO:0000793	condensed chromosome	79/2239	209/18122	3.53E-21	1.23E-18	1.01E-18	79
GO:0000775	chromosome, centromeric region	74/2239	188/18122	3.90E-21	1.23E-18	1.01E-18	74
GO:0098687	chromosomal region	107/2239	349/18122	5.68E-20	1.19E-17	9.80E-18	107
GO:0000779	condensed chromosome, centromeric region	53/2239	115/18122	3.67E-19	5.79E-17	4.76E-17	53
GO:0000776	kinetochore	53/2239	130/18122	2.64E-16	3.33E-14	2.74E-14	53
GO:0000777	condensed chromosome kinetochore	46/2239	103/18122	3.69E-16	3.88E-14	3.19E-14	46
GO:0005819	spindle	79/2239	301/18122	3.32E-11	2.99E-09	2.45E-09	79
GO:0005578	proteinaceous extracellular matrix	88/2239	359/18122	1.27E-10	9.62E-09	7.90E-09	88
GO:0044454	nuclear chromosome part	111/2239	493/18122	1.37E-10	9.62E-09	7.90E-09	111
GO:0005657	replication fork	27/2239	65/18122	3.28E-09	2.07E-07	1.70E-07	27
GO:0005874	microtubule	89/2239	389/18122	4.11E-09	2.35E-07	1.93E-07	89
GO:0000794	condensed nuclear chromosome	31/2239	88/18122	2.56E-08	1.34E-06	1.10E-06	31
GO:0031674	I band	40/2239	132/18122	3.53E-08	1.71E-06	1.41E-06	40
GO:0000940	condensed chromosome outer kinetochore	10/2239	12/18122	4.24E-08	1.91E-06	1.57E-06	10
GO:0000922	spindle pole	40/2239	135/18122	7.01E-08	2.94E-06	2.42E-06	40
GO:0044449	contractile fiber part	53/2239	204/18122	8.00E-08	3.15E-06	2.59E-06	53
GO:0042555	MCM complex	9/2239	11/18122	2.88E-07	1.07E-05	8.76E-06	9
GO:0005581	collagen trimer	29/2239	88/18122	3.69E-07	1.26E-05	1.04E-05	29
GO:0043292	contractile fiber	54/2239	219/18122	3.81E-07	1.26E-05	1.04E-05	54
GO:0030018	Z disc	35/2239	118/18122	4.39E-07	1.36E-05	1.12E-05	35
GO:0030016	myofibril	52/2239	209/18122	4.61E-07	1.36E-05	1.12E-05	52
GO:0030017	sarcomere	48/2239	187/18122	4.74E-07	1.36E-05	1.12E-05	48
GO:0005871	kinesin complex	21/2239	54/18122	6.83E-07	1.87E-05	1.54E-05	21
GO:0043596	nuclear replication fork	18/2239	43/18122	1.19E-06	3.13E-05	2.57E-05	18
GO:0005875	microtubule	38/2239	144/18122	3.51E-06	8.53E-05	7.01E-05	38

	associated complex						
GO:0072686	mitotic spindle	23/2239	68/18122	3.52E-06	8.53E-05	7.01E-05	23
GO:0098644	complex of collagen trimers	11/2239	20/18122	5.76E-06	0.000134	0.00011	11
GO:0051233	spindle midzone	13/2239	28/18122	9.30E-06	0.000209	0.000172	13
GO:0030424	axon	77/2239	385/18122	1.12E-05	0.00024	0.000197	77
GO:0030425	dendrite	89/2239	462/18122	1.14E-05	0.00024	0.000197	89
GO:0044297	cell body	92/2239	483/18122	1.31E-05	0.000265	0.000218	92
GO:0098794	postsynapse	80/2239	407/18122	1.48E-05	0.000292	0.00024	80
GO:0005583	fibrillar collagen trimer	8/2239	12/18122	1.67E-05	0.00031	0.000255	8
GO:0098643	banded collagen fibril	8/2239	12/18122	1.67E-05	0.00031	0.000255	8
GO:0097060	synaptic membrane	61/2239	291/18122	2.09E-05	0.000376	0.000309	61
GO:0000780	condensed nuclear chromosome, centromeric region	10/2239	19/18122	2.60E-05	0.000435	0.000358	10
GO:0043034	costamere	10/2239	19/18122	2.60E-05	0.000435	0.000358	10
GO:1902495	transmembrane transporter complex	65/2239	318/18122	2.62E-05	0.000435	0.000358	65
GO:0005813	centrosome	92/2239	492/18122	2.74E-05	0.000443	0.000364	92
GO:0034702	ion channel complex	59/2239	282/18122	3.00E-05	0.000473	0.000389	59
GO:0043204	perikaryon	29/2239	110/18122	4.91E-05	0.000755	0.00062	29
GO:1990351	transporter complex	65/2239	325/18122	5.27E-05	0.00079	0.000649	65
GO:0043025	neuronal cell body	80/2239	422/18122	5.54E-05	0.000811	0.000667	80
GO:0042383	sarcolemma	30/2239	118/18122	7.63E-05	0.001093	0.000899	30
GO:0045211	postsynaptic membrane	47/2239	220/18122	0.00011	0.001537	0.001264	47
GO:0030894	replisome	12/2239	30/18122	0.000124	0.001679	0.00138	12
GO:0000785	chromatin	86/2239	472/18122	0.000126	0.001679	0.00138	86
GO:0005911	cell-cell junction	76/2239	406/18122	0.000128	0.001679	0.00138	76
GO:0045121	membrane raft	57/2239	285/18122	0.000148	0.001883	0.001548	57
GO:0030496	midbody	34/2239	145/18122	0.000149	0.001883	0.001548	34
GO:0098857	membrane microdomain	57/2239	286/18122	0.000163	0.002008	0.001651	57
GO:0044420	extracellular matrix component	29/2239	119/18122	0.000221	0.002677	0.002201	29
GO:0005721	pericentric	9/2239	20/18122	0.000308	0.003661	0.003009	9

	heterochromatin						
GO:0005876	spindle microtubule	16/2239	52/18122	0.000365	0.004254	0.003497	16
GO:0043240	Fanconi anaemia nuclear complex	7/2239	13/18122	0.000379	0.004336	0.003565	7
GO:0043601	nuclear replisome	11/2239	29/18122	0.00041	0.004609	0.003789	11
GO:0044291	cell-cell contact zone	18/2239	63/18122	0.000444	0.00491	0.004036	18
GO:1990023	mitotic spindle midzone	6/2239	10/18122	0.000476	0.005169	0.00425	6
GO:0034703	cation channel complex	42/2239	203/18122	0.000503	0.005376	0.004419	42

Table S4: molecular function of GO analysis on DEGs.

ID	Description	Gene Ratio	Bg Ratio	p value	p.adjust	q value	Count
GO:0043142	single-stranded DNA-dependent ATPase activity	10/2104	12/16982	4.35E-08	4.45E-05	4.14E-05	10
GO:0015631	tubulin binding	67/2104	289/16982	2.17E-07	8.01E-05	7.45E-05	67
GO:0003777	microtubule motor activity	27/2104	77/16982	2.35E-07	8.01E-05	7.45E-05	27
GO:0003678	DNA helicase activity	20/2104	50/16982	7.68E-07	0.000196	0.000183	20
GO:0005539	glycosaminoglycan binding	49/2104	201/16982	1.98E-06	0.000404	0.000376	49
GO:0004386	helicase activity	39/2104	150/16982	4.13E-06	0.000704	0.000655	39
GO:0008017	microtubule binding	50/2104	217/16982	8.63E-06	0.00126	0.001173	50
GO:0016887	ATPase activity	82/2104	417/16982	1.25E-05	0.001595	0.001485	82
GO:0008094	DNA-dependent ATPase activity	23/2104	76/16982	2.91E-05	0.00331	0.003082	23
GO:0035173	histone kinase activity	9/2104	17/16982	6.45E-05	0.006592	0.006138	9
GO:0048407	platelet-derived growth factor binding	7/2104	11/16982	9.29E-05	0.008631	0.008036	7
GO:0003682	chromatin binding	88/2104	482/16982	0.000107	0.00913	0.008501	88
GO:0003774	motor activity	32/2104	132/16982	0.000125	0.009845	0.009166	32

Table S5: information of gene and lncRNA in each module.

Module	No. of Genes	No. of lncRNAs
turquoise	993	267
blue	714	166
yellow	125	73
green	139	31
black	43	73
magenta	62	4
red	108	15
purple	28	9
brown	80	314
greenyellow	26	10
pink	56	17

Table S6: Pathway enrichment analysis on gene sets in each module

Module	ID	Description	Gene Ratio	Bg Ratio	p value	p.adjust	q value	Count
turquoise	hsa04020	Calcium signaling pathway	28/359	182/7251	6.44E-08	1.71E-05	1.61E-05	28
turquoise	hsa04080	Neuroactive ligand-receptor interaction	33/359	278/7251	2.16E-06	0.000286	0.000269	33
turquoise	hsa04261	Adrenergic signaling in cardiomyocytes	21/359	144/7251	7.12E-06	0.000629	0.000592	21
turquoise	hsa04514	Cell adhesion molecules (CAMs)	19/359	145/7251	8.83E-05	0.004805	0.004523	19
turquoise	hsa04530	Tight junction	21/359	170/7251	9.07E-05	0.004805	0.004523	21
turquoise	hsa04924	Renin secretion	11/359	65/7251	0.000302	0.013326	0.012546	11
turquoise	hsa04022	cGMP-PKG signaling pathway	19/359	163/7251	0.000415	0.014679	0.013819	19
turquoise	hsa05414	Dilated cardiomyopathy	13/359	90/7251	0.000443	0.014679	0.013819	13
turquoise	hsa05410	Hypertrophic cardiomyopathy (HCM)	12/359	83/7251	0.000724	0.018102	0.017041	12
turquoise	hsa05412	Arrhythmogenic right ventricular cardiomyopathy (ARVC)	11/359	72/7251	0.000747	0.018102	0.017041	11
turquoise	hsa04024	cAMP signaling pathway	21/359	198/7251	0.000751	0.018102	0.017041	21
turquoise	hsa04270	Vascular smooth muscle contraction	15/359	121/7251	0.000875	0.01933	0.018198	15
turquoise	hsa04923	Regulation of lipolysis in adipocytes	9/359	54/7251	0.001188	0.024213	0.022794	9
turquoise	hsa04728	Dopaminergic synapse	15/359	130/7251	0.001829	0.034612	0.032585	15
turquoise	hsa05416	Viral myocarditis	9/359	59/7251	0.002257	0.039878	0.037541	9
turquoise	hsa04713	Circadian entrainment	12/359	96/7251	0.002621	0.043414	0.040871	12
blue	hsa04110	Cell cycle	37/260	124/7251	8.82E-25	1.95E-22	1.86E-22	37
blue	hsa03030	DNA replication	16/260	36/7251	1.82E-14	2.01E-12	1.91E-12	16
blue	hsa03460	Fanconi anemia pathway	17/260	55/7251	3.20E-12	2.36E-10	2.25E-10	17
blue	hsa03440	Homologous recombination	13/260	41/7251	8.64E-10	4.77E-08	4.55E-08	13
blue	hsa04115	p53 signaling pathway	13/260	69/7251	7.71E-07	3.41E-05	3.25E-05	13
blue	hsa00240	Pyrimidine metabolism	15/260	102/7251	2.96E-06	0.000109	0.000104	15
blue	hsa04114	Oocyte meiosis	15/260	124/7251	3.33E-05	0.001052	0.001002	15
blue	hsa03430	Mismatch repair	6/260	23/7251	0.000121	0.003344	0.003185	6
blue	hsa04914	Progesterone-mediated oocyte maturation	12/260	96/7251	0.00015	0.003674	0.0035	12
blue	hsa03410	Base excision repair	6/260	33/7251	0.000983	0.021715	0.020686	6
blue	hsa00230	Purine metabolism	15/260	175/7251	0.001484	0.029814	0.028401	15
green	hsa05150	Staphylococcus aureus infection	9/79	56/7251	6.80E-09	9.93E-07	7.59E-07	9
green	hsa04658	Th1 and Th2 cell differentiation	10/79	92/7251	4.62E-08	3.37E-06	2.58E-06	10
green	hsa04380	Osteoclast differentiation	11/79	128/7251	1.10E-07	5.35E-06	4.09E-06	11
green	hsa04659	Th17 cell differentiation	10/79	107/7251	1.97E-07	7.21E-06	5.51E-06	10
green	hsa05323	Rheumatoid arthritis	9/79	90/7251	4.71E-07	1.38E-05	1.05E-05	9
green	hsa05152	Tuberculosis	11/79	179/7251	3.21E-06	7.80E-05	5.96E-05	11

green	hsa04060	Cytokine-cytokine receptor interaction	13/79	270/7251	5.79E-06	0.000106	8.14E-05	13
green	hsa05321	Inflammatory bowel disease (IBD)	7/79	65/7251	5.83E-06	0.000106	8.14E-05	7
green	hsa05322	Systemic lupus erythematosus	9/79	133/7251	1.24E-05	0.000202	0.000154	9
green	hsa05140	Leishmaniasis	7/79	74/7251	1.39E-05	0.000204	0.000156	7
green	hsa04650	Natural killer cell mediated cytotoxicity	8/79	133/7251	9.06E-05	0.001203	0.000919	8
green	hsa04514	Cell adhesion molecules (CAMs)	8/79	145/7251	0.000166	0.001988	0.001519	8
green	hsa04612	Antigen processing and presentation	6/79	77/7251	0.000177	0.001988	0.001519	6
green	hsa04145	Phagosome	8/79	152/7251	0.00023	0.002394	0.001829	8
green	hsa05416	Viral myocarditis	5/79	59/7251	0.000427	0.004157	0.003177	5
green	hsa05330	Allograft rejection	4/79	38/7251	0.000727	0.006636	0.005071	4
green	hsa04062	Chemokine signaling pathway	8/79	185/7251	0.000857	0.00728	0.005564	8
green	hsa04620	Toll-like receptor signaling pathway	6/79	104/7251	0.000898	0.00728	0.005564	6
green	hsa05332	Graft-versus-host disease	4/79	41/7251	0.000973	0.007479	0.005716	4
green	hsa04940	Type I diabetes mellitus	4/79	43/7251	0.001167	0.008517	0.006509	4
green	hsa04142	Lysosome	6/79	123/7251	0.002135	0.014842	0.011343	6
green	hsa04621	NOD-like receptor signaling pathway	7/79	168/7251	0.002258	0.014984	0.011451	7
green	hsa05320	Autoimmune thyroid disease	4/79	53/7251	0.00255	0.016187	0.01237	4
green	hsa04640	Hematopoietic cell lineage	5/79	97/7251	0.003987	0.024255	0.018536	5
green	hsa04630	Jak-STAT signaling pathway	6/79	156/7251	0.006876	0.040158	0.03069	6
green	hsa05340	Primary immunodeficiency	3/79	37/7251	0.007411	0.041615	0.031804	3
magenta	hsa04974	Protein digestion and absorption	8/32	90/7251	3.39E-09	2.34E-07	1.85E-07	8
magenta	hsa04512	ECM-receptor interaction	6/32	82/7251	1.25E-06	4.30E-05	3.41E-05	6
magenta	hsa04933	AGE-RAGE signaling pathway in diabetic complications	6/32	99/7251	3.78E-06	8.70E-05	6.90E-05	6
magenta	hsa04151	PI3K-Akt signaling pathway	9/32	342/7251	1.11E-05	0.000192	0.000152	9
magenta	hsa04510	Focal adhesion	7/32	199/7251	1.98E-05	0.000274	0.000217	7
magenta	hsa04611	Platelet activation	5/32	123/7251	0.000181	0.002077	0.001648	5
magenta	hsa05146	Amoebiasis	4/32	96/7251	0.000781	0.007698	0.006106	4
magenta	hsa04610	Complement and coagulation cascades	3/32	79/7251	0.004919	0.042423	0.033654	3
red	hsa05322	Systemic lupus erythematosus	10/53	133/7251	3.06E-08	2.39E-06	2.26E-06	10
red	hsa05034	Alcoholism	10/53	180/7251	5.35E-07	2.09E-05	1.97E-05	10
red	hsa05203	Viral carcinogenesis	7/53	201/7251	0.000592	0.01539	0.014539	7
greenyellow	hsa04971	Gastric acid secretion	4/16	75/7251	1.75E-05	0.000735	0.000718	4
greenyellow	hsa04974	Protein digestion and absorption	3/16	90/7251	0.000921	0.019347	0.01891	3
pink	hsa04742	Taste transduction	3/18	83/7251	0.001042	0.048997	0.044992	3

Table S7: information of 15 hub genes.

Gene Symbol	ENTREZ ID	Gene Name
E2F3	1871	E2F transcription factor 3
DTL	51514	denticleless E3 ubiquitin protein ligase homolog
RBL1	5933	RB transcriptional corepressor like 1
NUSAP1	51203	nucleolar and spindle associated protein 1
ATAD2	29028	ATPase family, AAA domain containing 2
KIF18B	146909	kinesin family member 18B
MCM10	55388	minichromosome maintenance 10 replication initiation factor
RAD54L	8438	RAD54-like (S. cerevisiae)
NCAPH	23397	non-SMC condensin I complex subunit H
PLK4	10733	polo like kinase 4
DKC1	1736	dyskerin pseudouridine synthase 1
BUB1	699	BUB1 mitotic checkpoint serine/threonine kinase
CDCA5	113130	cell division cycle associated 5
KPNA2	3838	karyopherin subunit alpha 2
TBC1D31	93594	TBC1 domain family member 31

Table S8: GO and KEGG analysis for hub genes.

ID	Description	Gene Ratio	Bg Ratio	P value	Q value
hsa04110	Cell cycle	3/8	124/7250	0.00027	0.0054
GO:0000070	mitotic sister chromatid segregation	5/14	134/16992	5.35e-08	6.25e-06
GO:0007076	mitotic chromosome condensation	3/14	14/16992	1.61e-07	9.42e-06
GO:0000819	sister chromatid segregation	5/14	218/16992	6.05e-07	2.36e-05
GO:0098813	nuclear chromosome segregation	5/14	287/16992	2.35e-06	6.13e-05
GO:0030261	chromosome condensation	3/14	34/16992	2.624e-06	6.13e-05
GO:0007059	chromosome segregation	5/14	330/16992	4.65e-06	9.05e-05
GO:0007067	mitotic nuclear division	5/14	453/16992	2.16e-05	0.00036
GO:0071103	DNA conformation change	4/14	283/16992	6.61e-05	0.00091
GO:0033044	regulation of chromosome organization	4/14	288/16992	7.07e-05	0.00091
GO:0007063	regulation of sister chromatid cohesion	2/14	20/16992	0.00012	0.0014
GO:0007088	regulation of mitotic nuclear division	3/14	151/16992	0.00023	0.0025
GO:0051783	regulation of nuclear division	3/14	173/16992	0.00035	0.0034
GO:0007126	meiotic nuclear division	3/14	184/16992	0.00042	0.0037
GO:1903046	meiotic cell cycle process	3/14	195/16992	0.00049	0.0041
GO:0006323	DNA packaging	3/14	199/16992	0.00052	0.0041

Table S9: Clinicopathological characteristics of gastric cancer patients

	No. of Cases	%
**Total no.**	90	100
**Age**		
<67	40	44.4
≥67	50	55.6
**Gender**		
female	22	46.7
male	68	53.3
**Pathological types**		
adenocarcinoma	75	83.3
mucinous adenocarcinoma	5	5.6
signet ring cell cancer	10	11.1
**Pathological grade**		
<III	48	53.3
=III	42	46.7
**Diameter（cm）**		
<5.5	42	46.7
≥5.5	48	53.3
**Location**		
Upper third	17	18.9
Middle third	21	23.3
Lower third	52	57.8
**Tumor type**		
Borrman type Ⅰ/Ⅱ/VI	26	28.9
Borrman type Ⅲ	60	66.7
Early gastric cancer	4	4.4
**Serous membrane invasion**		
No	30	33.3
Yes	60	66.7
**Vascular or nerve invasion**		
No	66	55.4
Yes	24	44.6
**Lymph node metastasis**		
No	25	27.8
Yes	65	72.2
**AJCC stage**		
I/II	42	46.7
III/IV	48	53.3
**Overall survival**		
survived	30	33.3
dead	60	66.7
